# Unprecedented recent regional increase in organic carbon and lithogenic fluxes in high altitude Pyrenean lakes

**DOI:** 10.1038/s41598-023-35233-1

**Published:** 2023-05-26

**Authors:** Alejandra Vicente de Vera García, María Pilar Mata-Campo, Sergi Pla, Eduardo Vicente, Ricardo Prego, Matías Frugone-Álvarez, Josué Polanco-Martínez, Marcel Galofré, Blas Lorenzo Valero-Garcés

**Affiliations:** 1 Pyrenean Institute of Ecology, IPE-CSIC, 50059 Zaragoza, Spain; 2 Geological and Mining Institute of Spain, IGME-CSIC, 28003 Madrid, Spain; 3 CREAF, Campus UAB, 08193 Bellaterra (Barcelona), Spain; 4 Universitat Autonoma Barcelona, Bellaterra (Barcelona), 08193, Spain; 5 Instituto Cavanilles (ICBIBE), Universidad de Valencia, 46100 Burjassot, Spain; 6 Department of Oceanography, Marine Research Institute (IIM-CSIC), 36208 Vigo, Spain; 7 Facultad de Ciencias, Universidad Católica de la Santísima Concepción (UCSC), 4090541 Concepción, Chile; 8 Núcleo Milenio UPWELL, Concepción, Chile; 9 Economic Management for Sustainability (GECOS), IME, University of Salamanca, 37007 Salamanca, Spain; 10 Basque Centre for Climate Change-BC3, 48940 Leioa, Spain

**Keywords:** Carbon cycle, Limnology

## Abstract

We have conducted a monitoring survey and paleolimnological study of a W-E transect of six high altitude lakes (1870–2630 m asl) in the western and central Pyrenees (Spain) to evaluate the regional response to current global change in high altitude Mediterranean mountains. The reconstructed Total Organic Carbon (TOC_flux_) and lithogenic (L_flux_) fluxes during the last 1200 years show the expected variability as lakes differ in altitude, geological and climate settings, limnological properties and human impact history. However, all show unique patterns after 1850 CE, particularly during the Great Acceleration (after 1950 CE). Recent L_flux_ increase could be related to higher erodibility by rainfall and run-off during the longer snow-free season in the Pyrenees. In all sites, higher TOC_flux_ and geochemical (lower δ^13^C_OM_, lower C/N) and biological (diatom assemblages) signatures since 1950 CE suggest an increase in algal productivity, likely favored by warmer temperatures and higher nutrient deposition. These recent, unprecedented L_flux_ and TOC_flux_ increases, in spite of their diverse history and limnological properties of the lakes, demonstrate the regional impact of the Great Acceleration not only in the ecological dynamics of alpine lakes but also in the hydrological cycle in high altitude mountain watersheds.

## Introduction

Lakes are a central component of the carbon cycle and several paleolimnological studies have shown organic carbon burial rate increases during the last centuries, although the causes for regional variability, the specific involved processes, and likely future scenarios remain uncertain^1,2^. At a global scale, recent changes in mountain lake dynamics have been related to climate variability, direct human impact in the watersheds^3–5^ and increased global deposition rates of nutrients^6^, altering their structure, functioning and diversity^7^ and causing eutrophication and pollution^8,9^.

To assess the recent rates of response and vulnerability of ecosystems and territories to rapid shifts of the Earth System they have to be evaluated in the context of the Anthropocene^10^ and the current Great Acceleration^11^ (GA). In particular, Mediterranean mountains face great environmental risks as temperature and precipitation regimes^12^ and human pressure^4,5,13^ have changed during the last century. A clear example of such trends are documented in the Pyrenees where, since 1959, the annual mean temperature has increased by more than 1.6 °C and summer temperature by more than 2.3 °C. The decrease in annual precipitation has been small (-2.5%, last 50 years) but winter rainfall events are now more frequent^14^ .The high altitude lakes in the Pyrenees provide an opportunity to investigate the role of climate change and human activities in carbon and sediment dynamics, as past climate variability^5,15–23^ and the history of human impacts are relatively well known^24^. Human activities had a restricted impact till the onset of ca.twelfth century deforestation phase^24,25^. The abandonment of traditional agropastoral activities has led to large socioeconomic changes since the mid twentieth century, and currently, lakes provide numerous services for new infrastructure development for energy resources, ski resorts and tourism^24,26^.

**Figure 1:**
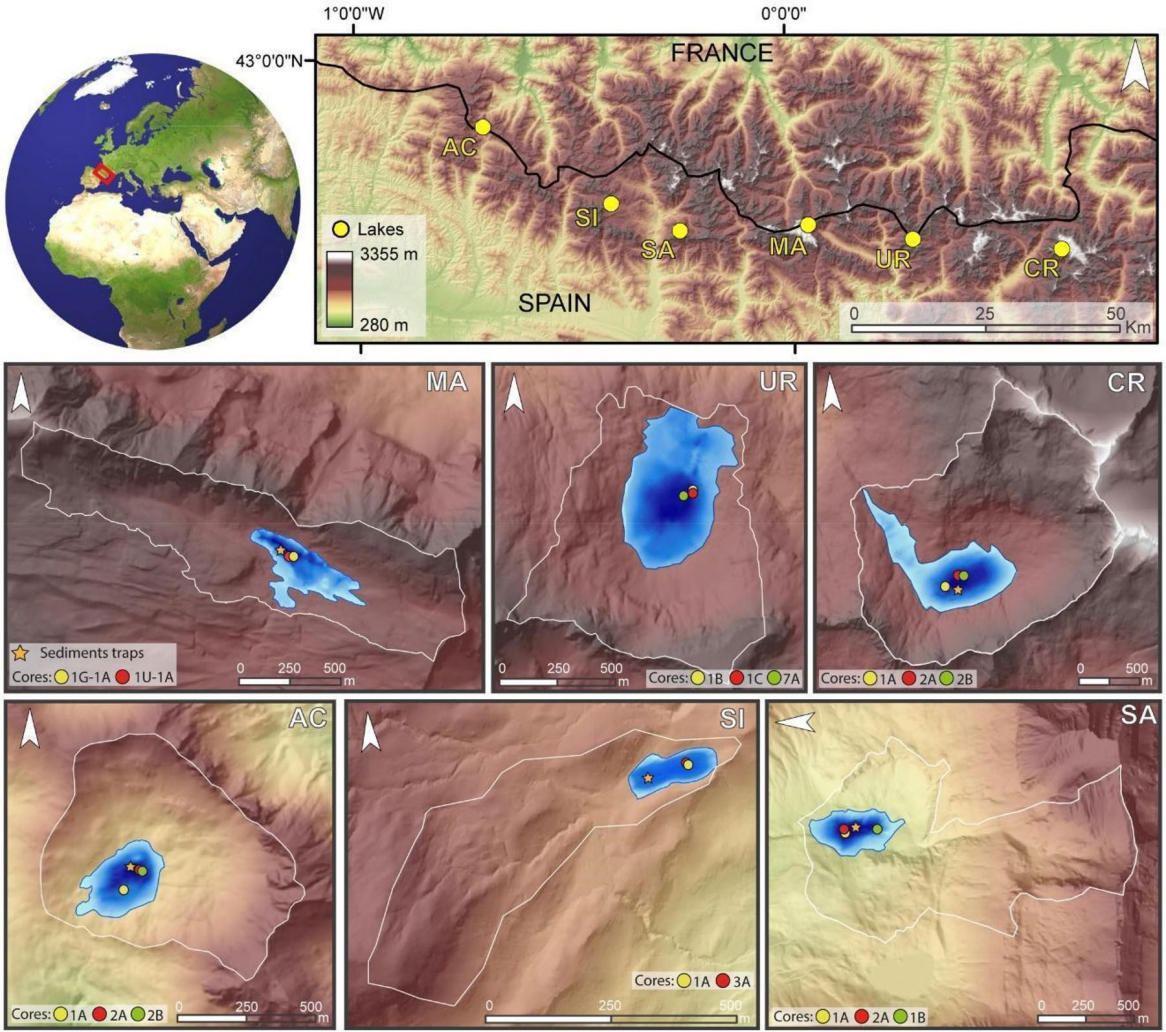
(**A**) Location of Pyrenean Lakes and the watersheds included in this study. (**B**) Bathymetry of the lakes, location of the cores and sediment traps. Bathymetric legend as in Fig. S1.

Some of the observed hydrological changes caused by recent climate change in the Pyrenees include melting glaciers and decreased snow cover^27^, increased sediment dynamics^28^ and ecological changes in alpine lakes^4,5^. However, the absence of long-term series and high-resolution reconstructions along geographic transects have impeded the evaluation of the regional nature of these recent observations within the context of past periods of rapid change. To investigate the nature of recent changes in high altitude Pyrenean watersheds, we reconstruct past organic carbon and depositional dynamics in six high altitude (1870–2630 m asl) lakes along a West–East transect in the Pyrenees (Fig. 1): Acherito (AC), La Sierra (SI), Sabocos (SA), Marboré (MA), Urdiceto (UR), and Cregüeña (CR). The selected lakes reflect the variety of Pyrenean lakes in terms of climate, geology, limnological properties and human impact. We evaluate their response during the last 1.2 ka to climate and human impact, and characterize the uniqueness of the Great Acceleration against the backdrop of the Anthropocene.

## Study sites

The six lakes have been monitored, including annual water and sediment composition and hourly temperature profiles for 5 years^14,29^. The selected lakes represent an altitudinal transect, from 2633 m asl (CR) to 1877 m asl (AC), with variable sizes (the largest CR, 0.445 km^2^, the smallest SI, 0.013 km^2^) and depths (7 m, SI; 99 m, CR). The watershed surface areas range between 3.4 km^2^ (CR) and 0.57 km^2^ (AC) and the geology includes mostly carbonate (AC, MA, SA) or siliceous (CR, UR, SI) formations. All lakes are of low productivity (ultra-oligotrophic to mesotrophic) and dimictic (a first overturn period after winter inverse stratification and a second overturn period after summer stratification) with different alkalinity (13.9 ppm in CR to 141.6 ppm in SA) and conductivity (14 µS cm^−1^ in CR to 209 µS cm^−1^ in SA) (Table S.1). Historic human impact in the watersheds is varied. Medieval deforestation for grazing occurred in the upper Gállego (SI, SA) and Aragón Subordán (AC) valleys. UR was dammed in the 1930s and water depth increased from < 10 m up to 25 m. A dam was also built in the 1940s in MA, but it was decommissioned early on, and it did not affect the lake level. SA is close to a ski area built in the 1980s. All lakes are located above the tree line and vegetation cover is scarce. Although all originated after the last deglaciation, glacier expansion documented by moraine deposition during the Little Ice Age (1200–1850 CE) only occurred in the CR watershed^30^. MA watershed is not connected to the Monte Perdido Glacier, but documentary evidence showed a smaller snow and ice accumulation in the NW shore till the mid 1950s^31^. Bathymetry, watershed topography, geology and core location of all lakes are shown in Fig. S.1 and Table S.1.

## Results

### Altitudinal transect of lake depositional systems

The sedimentary sequences have been analyzed using a multiproxy approach including detailed sedimentological, compositional, geochemical, and isotope analys es. Age models based on ^210^Pb, ^137^Cs, and ^14^C dates were obtained with Bayesian age-depth modeling^32,33^ (Figs. S.3, S.4.A, B, Table S.3). The sedimentary sequences of the lakes with the main proxies are shown in Figure S.2. A, B, C. In AC, SI, CR and UR, deposition is dominated by silt facies with some intercalated coarser facies, more frequent during the Little Ice Age (LIA) and with finer, more organic-rich facies since 1850 CE. In MA, rhythmite facies deposited during the last two millennia, reflect the dominance of seasonal ice cover dynamics. Dam construction in the early 1930s in UR favored finer deposition in the profundal area since then; however, no significant impact was detected in MA after dam construction in the 1940s. The CR sequence comprises an intercalated 7 cm thick fining upward layer, interpreted as a co-seismic turbidite caused by the largest regional earthquake during the last millennium (the Ribagorza earthquake, 1375 CE). The SA sequence is the only one with carbonate facies, but it also shows coarser silts during the LIA and finer silts since then. In this lake, a depositional change occurred after the 1980s, with more carbonate–rich facies in the littoral areas and finely laminated facies in the deepest areas, suggesting the expansion of the charophyte and macrophyte littoral meadows, increased primary productivity, and dominant anoxic conditions in the profundal areas.

### Lithogenic flux, organic carbon flux and depositional and productivity proxies

**Figure 2:**
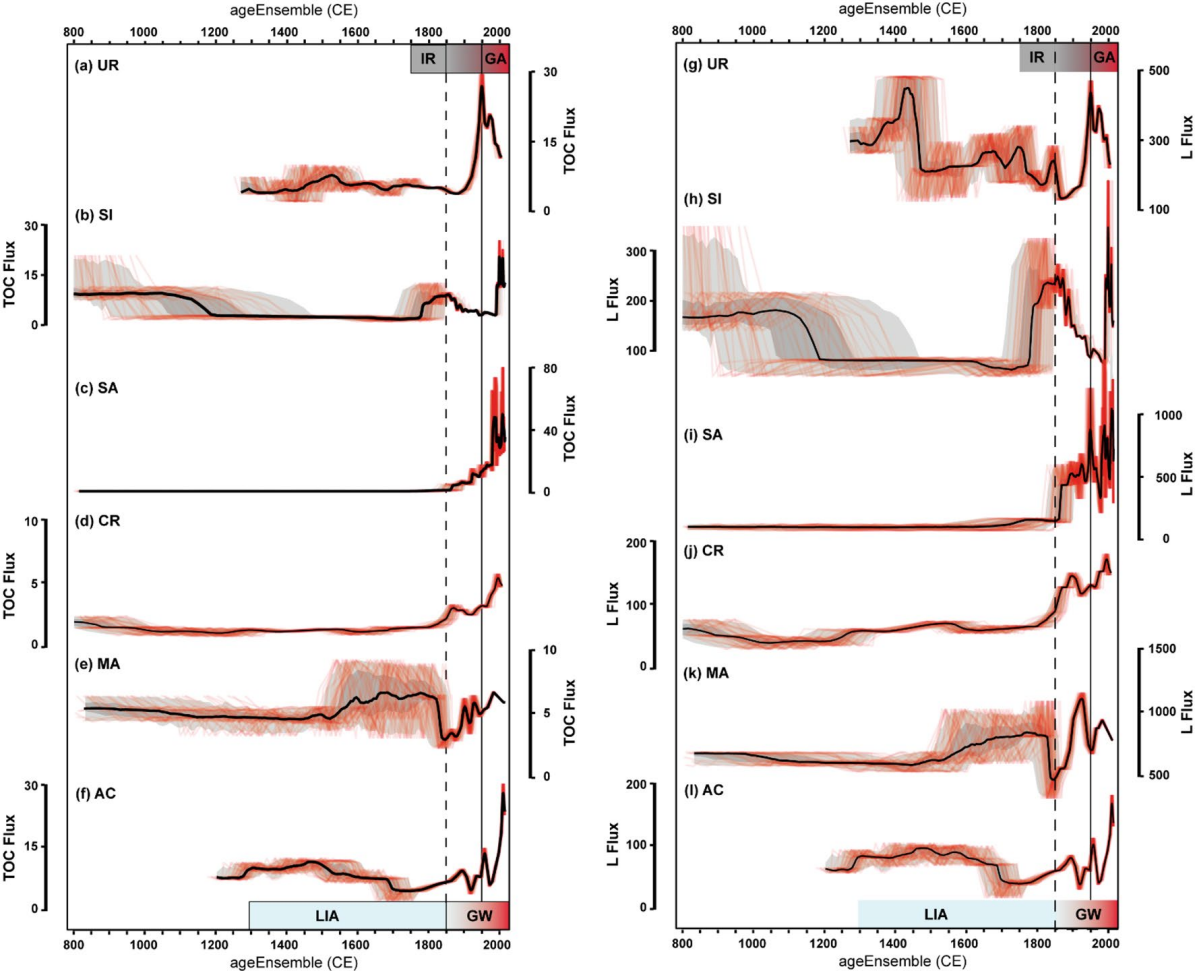
Lithogenic and TOC fluxes for the last 1200 years in the studied lake sequences. The climate phases (Little Ice Age, LIA and Recent Global Warming, GW) and the global change phases (Industrial Revolution, IR and Great Acceleration, GA) are also indicated. The median estimate is shown in black, and the 50 % and 95 % highest-probability density regions are shown in dark and light gray. Random age-uncertain ensemble members are shown in red.

Lithogenic fluxes (Lflux) calculated from sediment traps are between 20 and188 g m^−2^ year^−1^ in silicate-dominated basins, and higher in carbonate basins (up to 544 g m^−2^ year^−1^ in MA). Fluxes calculated from the first cm of the sediment cores range from 132 to 219 g m^−2^ year^−1^ in lakes located in silicate watersheds (CR, UR, SI, AC), and they are larger in carbonate-dominated watersheds (1274 in SA, 772 g m^−2^ year^−1^ in MA). Flux trends calculated from the sediment sequences directly reflect the age models, and consequently they have variable uncertainties and time-resolution during the last 1200 years. L_flux_ plots with the associated time uncertainty (Fig. 2) show large variability for the last 1200 years. In this study, we focus on the last 200 years, as the age models for our sequences are more robust for this period. L_flux_ for SI shows a high value pre-LIA phase coherent with the medieval human impact in the Gállego valley^24^. UR and AC have moderate to high L_flux_ during the first part of the LIA. Both MA and SI have high sediment fluxes prior to the end of the LIA, contrary to AC and UR. SA and CR do not show significant changes prior to 1850 CE. In spite of this variability, all lakes show a change in L_flux_ patterns after 1850 CE and the highest L_flux_ at some point during the last 200 years. SI values increased at the end of the LIA and since 1950, but lower values occurred during 1850–1950. MA shows an abrupt decrease at the end of the LIA and a recovery in the twentieth century. UR fluxes were the highest during dam construction (ca. 1940) but the values remained high during the last decades. In spite of the different nature of the depositional processes and the expected variability among all lakes, late twentieth century L_flux_ values were among the highest fluxes observed in all records.

The periods with higher sediment fluxes prior to 1850 CE are coherent with changes in sedimentary facies, as they correspond with coarser sediments and more frequent coarser layers deposited during some phases of the LIA. To obtain a geochemical proxy of sediment input we calculated a “Compositional PCA” (PCA_comp_) including TOC, TIC, geochemical (all elements measured by ICP-OES) and grain size (%sand) data (Fig. S.2A–C). The first PC1_comp_ explains up to 53% of the variance. We interpret this first principal component as related to changes in the lithogenic composition of the sediments. Lakes at lower altitude show a relatively greater detrital component in the sediments before 1850 CE, followed by an abrupt increase in the organic component, particularly marked since the mid twentieth century. The two lakes at higher altitudes show somewhat different patterns: CR trends are similar, but with smaller ranges, and in MA, PC2_comp_ better reflects the silicate component, although it only explains 25% of the total variance.

The TOC_flux_ based on sediment trap data ranges between 2.8 g m^−2^ year^−1^ (CR) to 46 g m^−2^ year^−1^ (AC), and the values are similar to those calculated from core-top sediment samples (from 4.8 g m^−2^ year^−1^ in CR to 49 g m^−2^ year^−1^ in SA). Sediment traps have higher TOC content and lower C/N (5–15) and δ^13^C_OM_ (−33‰ to –25‰) values than surface and downcore sediment samples. The TOC content in the lake sediments ranges between 0.06 (MA) and 13% (AC). TOC_flux_ and L_flux_ trends are similar, with generally lower TOC_flux_ values prior to 1850 CE, and the most prominent changes associated to the LIA and the GA. TOC_flux_ is high in early medieval times (SI) and during some phases of the LIA (AC, MA, UR). The 1850 CE transition is marked by an increase in TOC_flux_ in three lakes (AC, CR, SA), a decrease in MA and SI and no changes in UR. However, values during the GA are the largest in all cases. The largest increase in TOC_flux_ occurred in the 1970s in SA (from 14 to 66 g m^−2^ year^−1^) and even though it has decreased afterward, still remained high (> 40 g m^−2^ year^−1^). In SI, the largest change occurred around 1980 CE (from 4 to 12 g cm^−2^ year^−1^). In CR, after a small increase in TOC_flux_ around 1850 CE, it also intensified during the GA (from 3.1 to 4.9 g m^−2^ year^−1^). MA is the only lake with similar values during the GA (4.5 to 6.5 g m^−2^ year^–1^) and the LIA (2 to 9 g m^−2^ year^−1^). The highest values in UR occurred after the damming in the 1940s (from 12 to 31 g m^−2^ year^−1^) and although later decreased, still remained at relatively high values (Fig. 2).

The TOC_flux_ trends are coherent with other organic proxies such as C/N molar ratio, δ^13^C_OM_ in bulk organic matter, diatoms, biogenic silica (BioSi) and chlorophyll. Our survey shows that soil and watershed vegetation have higher C/N values with high variability (12–70) and a relatively small δ^13^C_OM_ range (−30‰ to −25‰), while lake sediments and Particulate Organic Matter (POM) samples have the lowest C/N ratios (mainly from 6 to 15, except for SA sediments that reach values of up to 35) (Fig. S.6). CR has the highest C/N values and MA the lowest. The complexity of organic matter sources^34^ is illustrated by the varied trends in C/N ratios in the lakes along the last 1200 years and particularly during the last two centuries. However, compared with pre-industrial values, C/N values are lower in the twentieth century and some show decreasing trends (UR, CR, SI, AC), except in carbonate-watershed lakes with no trend (MA) or increasing values (SA).

The lowest δ^13^C_OM_ values correspond to sediment traps (−33‰ to −25‰), lake sediments (−26‰ to −17‰) and POM (−30‰ to −25‰). Aquatic and terrestrial vegetation have similar δ^13^C_OM_ values, but aquatic samples have relatively lower C/N values than terrestrial, although the range is large (10–50). Soil samples have lower C/N values (12–23) than terrestrial and aquatic vegetation, but similar δ^13^C_OM_ signatures as sediment and vegetation samples. Sediments collected in the creeks feeding the lakes have relatively low C/N values (5–20) and more positive δ^13^C_OM_ (−26‰ to −17‰). A sample from a biofilm in CR buoy has the highest δ^13^C_OM_ value (−18‰). Most lake sediment samples—except SA—plot in the same δ^13^C_OM_ (−32‰ to −20‰) vs C/N (7–15) graph, but each lake has a distinctive distribution (Fig.S.6). Higher altitude lakes have relatively higher δ^13^C_OM_ (UR > MA > CR) than lower altitude sites. SA samples have the largest C/N (5 to 35) and δ^13^C_OM_ ranges distributed in two distinct populations with some of the highest δ^13^C_OM_ values (up to −7‰). Among the other sites, small, shallow SI has the largest δ^13^C_OM_ range (−32‰ to −26‰). MA and UR also have large C/N ranges and among the highest δ^13^C_OM_ ranges (−27‰ to −21‰). CR and SI have relatively more negative δ^13^C_OM_ values (−29‰ to −25‰). In spite of the δ^13^C_OM_ variability in each lake during the last 1200 years (Fig. S.2A–C) all time series (except MA) show a conspicuous decrease trend starting mid nineteenth century (end of the LIA) and intensifying since the mid twentieth century. In CR, the δ^13^C_OM_ decrease is about 1‰ associated with rather constant C/N values. UR had a larger δ^13^C_OM_ decline (−6‰) but started earlier (around 1700 CE) with an increasing C/N till 1950 and decreasing afterwards. SI also has a large δ^13^C_OM_ decline (−6‰) associated with variable but decreasing C/N values. AC showed a −2‰ decrease with lower C/N values during the GA. Carbonate lakes showed a different behavior: SA had an increasing δ^13^C_OM_ (6‰) during the IR and a large decline with abrupt shifts (up to 20‰) during the GA. MA also showed increasing δ^13^C_OM_ (up to −4‰) between 1850 and 1950 and then decreasing during the GA with little changes in C/N. Some of the observed decline in δ^13^C_OM_ during the last two centuries could be due to the documented decreasing in δ^13^C_OM_ value for atmospheric CO_2_ from pre-industrial (−6.4‰) to recent times (−8.4‰ in 2014) (“Suess effect”)^35^. However, as the δ^13^C_OM_ range in our data set is larger (from 1 in CR, to 20‰ in SA) it is likely that other factors (OM sources, changes in productivity rates, and exchange with atmosphere)^36,37^ have contributed to these recent patterns.

Diatom assemblages have been analyzed in surface sediments of all lakes and four core sequences (AC, MA, SA and CR), although diatoms were poorly preserved in MA. The CR, AC and SA diatom records show recent rapid changes in diatom assemblages. All three records started with benthic and thycoplanktonic taxa dominance, but all three lakes show an unprecedented recent increase in small planktonic diatoms (*Cyclotella* spp., *Discotella* spp.,* Pantocsekiella* spp. and planktonic *Fragilaria* spp.) (Fig. S7.A–C). Biogenic silica and chlorophyll content have been measured in AC and CR sequences (Fig. S.7.A–C). Both have lower values in the high altitude, silicate watershed site (CR < 1.5% BioSi, 18 ppm chlorophyll) and higher in the intermediate altitude AC (BioSi up to 6% and 337 ppm chlorophyll). Both sites show increasing BioSi since 1950 reaching the highest values in the records at the top of the sequence. Chlorophyll concentrations have also been higher since 1950, but there were similar prior peaks in CR and even with larger values in AC (Fig. S.7.A–C).

### Change points in time series

**Figure 3:**
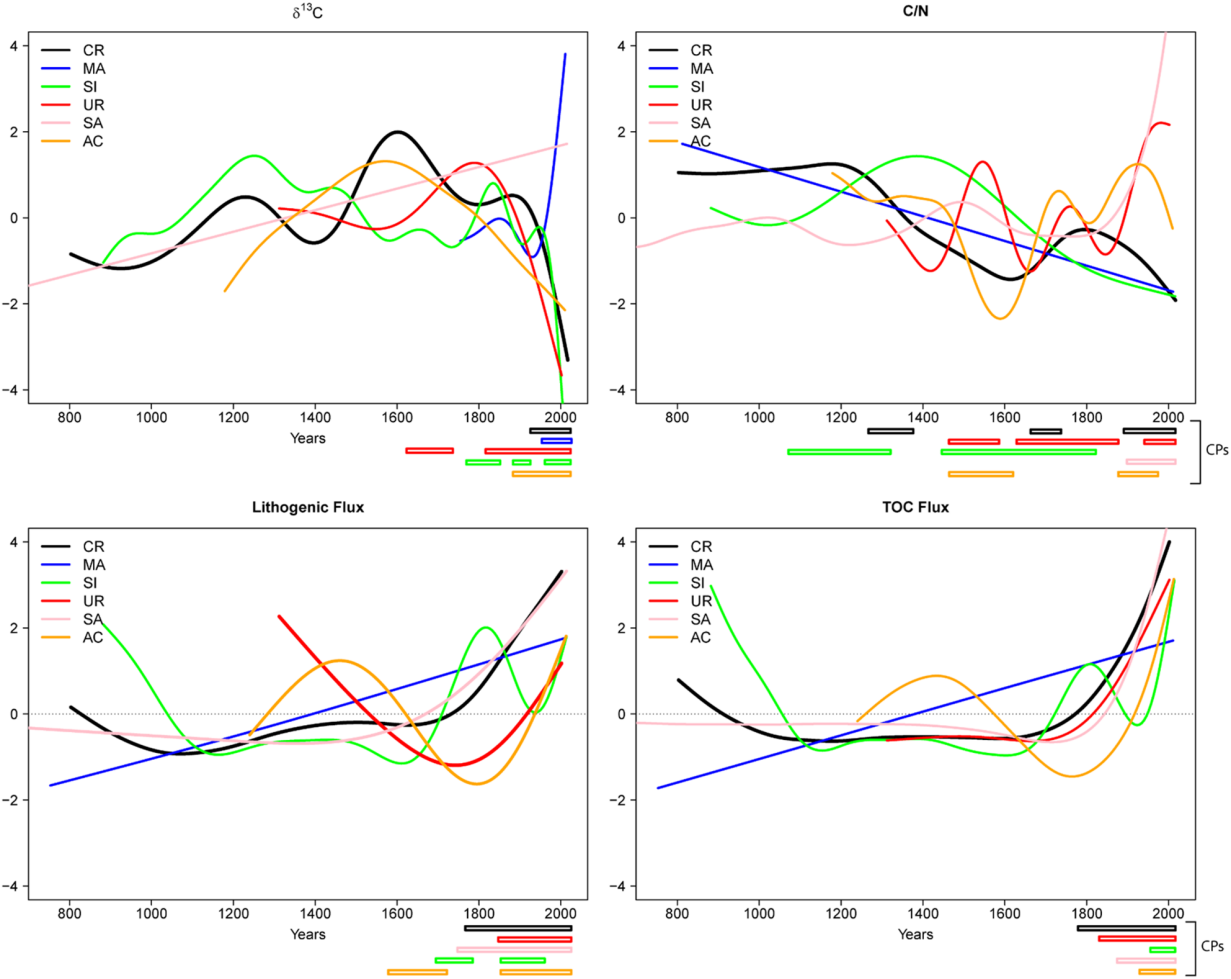
Results of the GAM trends fitted to Lflux, TOC flux, C/N and δ^13^C_OM_ time series and location of the main periods of significant temporal changes. The first derivatives and 95% simultaneous confidence intervals of the first derivatives are in Figs. S.9 and S.10.A,B).

**Figure 4:**
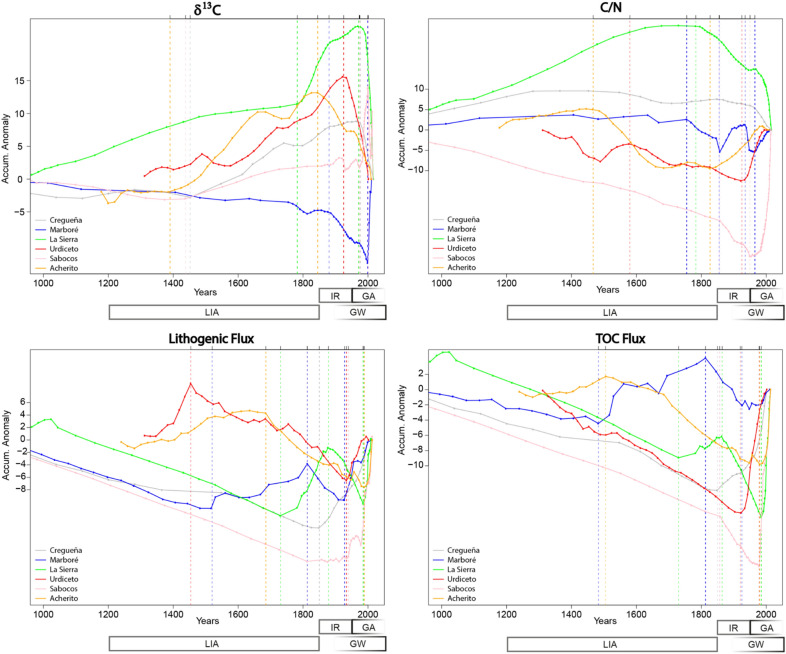
Accumulated anomalies from the mean value of each variable (δ^13^C_OM_, C/N, Lflux, TOCflux) obtained through the CumSum method. Vertical lines indicate the years when changes in trend occur.

Identification of potential Change Points (CP) in our time series using statistical tools is hampered by the non-regular nature of the data and the relatively low number of elements. We applied two methodologies to the L_flux_, TOC_flux_, C/N ratio and δ^13^C_OM_ time series, one based on GAMs (Generalized Additive Models)^38,39^ (Fig. 3) and other on Cumulative Sum (CumSum)^40^ (Fig. 4). Both are statistical models intended to estimate nonlinear trends and to identify periods of significant temporal changes (or simply change points –CP) in unevenly spaced paleoenvironmental time series. Each technique has pros and cons in terms of the identification of CPs, the quality and readability of graphic outputs.

Both GAM and CumSum identified several CPs prior to 1850 and some during the twentieth century. The CumSum (Fig. 4) L_flux_ plots show main L_flux_ CPs in the nineteenth century (1850 CR, 1813 MA, 1878 SI, 1813 SA, 1885 AC) and in the twentieth century (1928, MA; 1925 and 1935, UR; 1940 and 1985, SA; 1989 AC). The GAM analysis (Fig. 3) identified the most significant CPs during the twentieth century, around the mid nineteenth century (end of the LIA) and some smaller in the previous centuries (CR, ca. 1750; SI, ca.1670–1790 and ca. 1830–1900; UR, around 1870; SA, around 1750; AC, around 1570–1700 and 1850).

**Figure 5:**
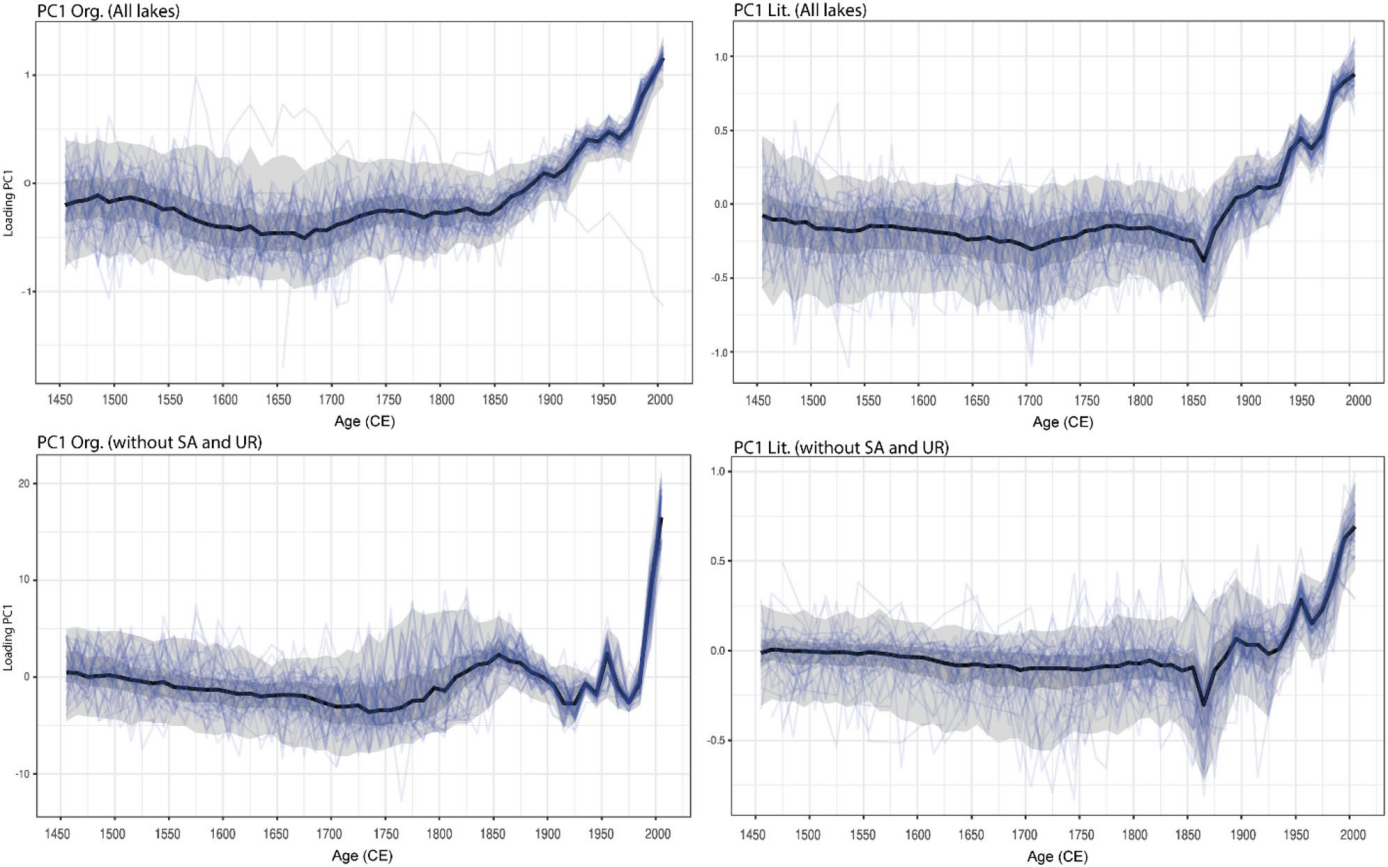
Principal component analysis for all proxies from all lakes datasets (left) and excluding SA and UR (right) of (top) organic matter variables (C/N, δ^13^C_OM_ and TOC fluxes) and (bottom) sediment delivery (Lithogenic fluxes and “compositional PC1” (see methodology and Fig. S2). The median ensemble member is shown in black, with the 50% and 95% highest-density probability ranges shown in dark and light gray, respectively. Age-uncertain organic matter and sediment delivery ensemble members are shown in blue.

**Figure 6:**
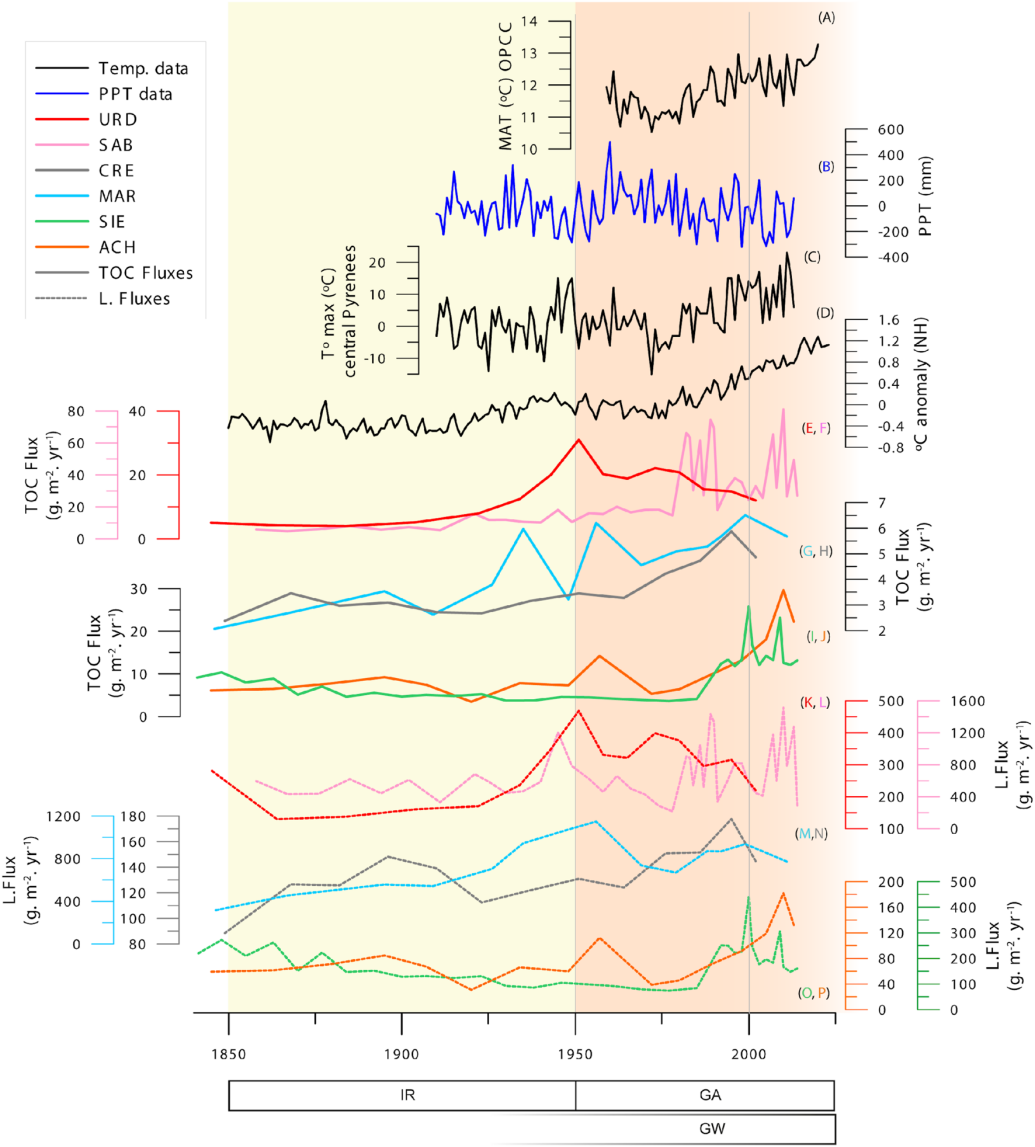
Lithogenic fluxes, TOC fluxes and climate reconstructions based on instrumental data since 1850 CE. (**A**) Mean annual temperature in the Pyrenees obtained by OPCC (https://www.opcc-ctp.org/sites/default/files/documentacion/en_informe_opcc_adapyr.pdf), (**B,C**) regional climate series of precipitation and temperature over the central Pyrenees between 1910 and 2013 (Pérez-Zanón, 2017^51^) and (**D**) gridded temperature anomaly averages for the northern hemisphere from the HadCRUT global temperature dataset (Morice et al. (2021^49^); Osborn et al. (2021)^50^). (**E–J**) TOC fluxes and (**K–P**) lithogenic fluxes. Lakes are grouped into (1) more anthropized (UR in red and SA in pink), (2) higher altitude (CR in gray and MA in blue) and (3) lower altitude (SI in green and AC in orange). Climate phases (Recent Global Warming, GW) and global change phases (Industrial Revolution, IR and Great Acceleration, GA) are also indicated.

To understand the temporal evolution associated with the range of variability in lake age-depth models in terms of organic matter accumulation and sediment input, we implemented two main component analyses of uncertain age (PC_AU_) using two sets of variables^41^): (1) TOC_flux_, CN, and δ^13^C_OM_ that were interpreted as indicators of organic matter accumulation and sources (PC_AUorg_) and (2) L_flux_ and PC1comp that represent sediment delivery to lakes (PC_AUlit_) (see Fig. 5 and methodology). The two first principal components (PC1_AUorg_ and PC1_AUlit_) explained 52% and 49% of the variance. This means that for the same age assemblage, more than half of the variability in the data associated with sediment and organic matter input increases simultaneously. Figure 5 shows that the main changes for all lakes occurred around 1850, 1900 and 1950 CE, both in terms of a rapid increase in organic matter accumulation and sediment flux into the lakes. We run similar PCA analyses excluding UR and SA time series,—the two lakes that could have been more affected by dam and ski resort construction, respectively—and the results are comparable (Fig. 5), although the % of variance explained by the first components changed: 68% for PC_AUorg_ and only 26% for PC_AUlit_. The PC1_AUorg_ y PC1_AUlit_ versus age plots show similar structure, with larger positive values around the end of the LIA (1850), 1950 and recent decades (see Supplementary Material for details of both PCAs, Fig S.5). Interestingly, the PC1_AUorg_ variability was larger if SA and UR were not included in the data and positive peaks were more clearly associated to warmer periods at the end of the LIA and during the twentieth century (Fig. 6 and references). However, we note that the time at which the loadings exceed the 95% probability density region (blue region in the Fig. 5) coincides with the timing when both trends became faster and exponential after 1950 CE.

## Discussion

### Variability in depositional evolution and lithogenic fluxes

The different timing and intensity of sediment input variability may reflect local responses of the watersheds and lakes to varied climate and human pressure in mountain environments during the last millennium^13,24,42^. Periods with higher L_flux_ during medieval times in lower altitude lakes (SI, between 800 and 1200 CE; AC, between 1300 and 1500 CE) (Fig. 2) and the site located close to a mountain pass (UR, 1300–1500 CE) (Fig. 2) could be related to the deforestation for summer grazing pastures that started in the twelfth century with the socio-economic changes brought by the expansion of the Aragon Crown^24^. During the LIA (1300–1850 CE) the human presence in higher altitude areas was reduced^21,25^, and another peak in human population was reached after the mid nineteenth century. Rural exodus since the mid twentieth century again decreased human pressure in mid mountains^28^.

At higher altitude sites, sediment fluxes are mostly controlled by hydrological and cryospheric processes, as snowmelt is responsible for mobilization of sediment in the watershed and deposition in the lakes. Monitoring data in the Izas experimental watershed^43,44^ and reconstructions of past cryospheric activity^44,45^ have shown that in the Pyrenees, periods with more precipitation—either as snow in winter or rainfall the rest of the year—and higher seasonality (rapid spring–summer snowmelt) are conducive to higher run-off, erosion and sediment delivery in the watersheds. On the other hand, during colder episodes lake bioproductivity is reduced^20–22,42,44,46^. The maximum extent of LIA glaciers in the Pyrenees occurred during the last decades of the seventeenth century (Maunder Minimum) minor re-advances between 1750 and 1800, and a significant expansion until 1830 (Dalton Minimum)^47^. In our records we have identified periods with higher L_flux_ and higher bioproductivity that we relate to dominantly wetter and warmer conditions respectively. L_flux_ time series show that greater sediment delivery did not occur during the coldest periods as the Maunder minimum (1620–1715 CE), but prior to 1570 CE and after 1715 CE (Fig. 2). We do not expect L_flux_ variability to follow global regional glacier expansion and retreat during the LIA in the watersheds that have been glacier-free during the last millennia (AC, SI, SA, UR). In MA and CR several moraines show active glaciers during the LIA, but the Equilibrium Line Altitudes (ELA) fluctuated at higher elevations, between 2620 and 2945 m asl and the glacier ice was never in contact with the lakes^42^*.*There are historical reports from eighteenth century Pyrenean explorers of a small ice accumulation reaching the NW margin of Marboré Lake that lasted till early twentieth century^31^ This smaller ice patch could have been responsible for the important increase in sediment input during the second phase of the LIA (1750- 1850 CE) in Marboré sequence.

The second large increase in L_flux_ during the last 1200 years occurred during the last century and particularly during the last decades. This is somehow unexpected as the watersheds have not been directly affected by large land use changes or direct human activities and changes in precipitation amount have been relatively small. Since the 1950s, agropastoralism pressure in the Pyrenees has been greatly reduced, and new socioeconomic activities (tourism) have become the main agents of landscape transformation^46^. In absence of direct changes in land cover, we propose that new precipitation regimes could be responsible for this trend. The data from the high altitude Izas experimental basin—including SI watershed—^44^ shows the paramount relevance of the short (two-month) snowmelt period as it is responsible for about 50% of the total annual runoff and 35–43% of the total solute and suspended sediment yield. Higher temperatures also increase the probability of larger runoff events in spring and more frequent rain events with higher erodibility impact than snow^48^. The reconstructions and instrumental temperature data for central Pyrenean area (Fig. 6) show the recent increase in annual mean temperature and the variability of precipitation^14,49–51^.The recent increases in L_flux_ in most sites suggest these regional changes in precipitation seasonality and hydrology are affecting sediment transport and delivery simultaneously across the Pyrenees at various altitudes.

### Variability in organic matter fluxes

Organic accumulation in high altitude Pyrenean lakes is limited by the reduced vegetation and soil cover in their watersheds and by the low productivity, which strongly depends on seasonality (ice-free season)^5,15^. Relatively low C/N ratios (7–15) suggest dominant algal sources in lake production, and variable contribution of terrestrial carbon. Periods with increased organic carbon accumulation may respond to higher input of watershed-derived organic matter (higher C/N) during periods of higher erosion and/or run-off and/or increased lake bioproductivity (lower C/N, higher BioSi, chlorophyll). The C/N records have a large variability, but during the GA, all those located in silicate—watersheds showed decreasing trends, indicative of increasing lake productivity. MA values have remained constant during the last centuries and SA is the only one with an increasing trend, likely related to mac rophyte development in the littoral areas. An increase in BioSi and chlorophyll and changes in diatom assemblages also occurred in CR, SA and AC during the last decades (Fig. S.7A–C). As in most studies^34^, our watershed-lake survey shows that aquatic and terrestrial vegetation have similar δ^13^C_OM_ range and lake sediments and POM have lower values (Fig. S.6). Changes in productivity greatly affect δ^13^C_OM_ values^34,52^ as during carbon uptake, phytoplankton preferentially assimilates the lighter isotope ^12^C^36^. Under conditions of low to moderate primary productivity, plankton preferentially uptake the lighter ^12^C, resulting in lower δ^13^C_OM_ values^52^. The δ^13^C_OM_ shows a large decrease at the end of the LIA and the onset of the Industrial Revolution (IR) and reached the most negative values during the last decades. The two lakes with carbonate-facies and dominant carbonate formations in the watersheds (MA and SA) show a distinctive large increasing δ^13^C_OM_ trend in recent times, which points to other sources of DIC in these lakes. In MA, more DIC from carbonate bedrock could reach the lake during warmer periods^42^. In SA, recent development of charophyte meadows has shifted the type of organic matter accumulation and contributed to higher δ^13^C_OM_. The ice-cover period could be another factor as shorter ice cover decreases the accumulation of CO_2_ during winter in the water column, which also decreases CO_2_ exchange between the lake and the atmosphere leading to more positive δ^13^C_OM_^15^.

Prior to the unprecedented recent increase in the diatom planktonic component that reflects an increase in lake productivity, the tichoplanktonic taxa were abundant in CR, AC and SA. In CR, the deepest lake, *Aulacoseira* spp. showed higher abundance prior to the mid twentieth century (Fig. S.7.A–C). These species are very abundant during spring and autumn overturn periods in dimictic lakes. Hence, their recent decrease suggests shorter overturn and longer summer stratification periods^22^. In lake AC small fragilaroids dominate the tychoplankton, and the abundance of these species is sensitive to shoreline water turbulence and temperatures^53^. In lake SA, the small fragilaroid *Pseudostaurosira cf. robusta* also showed a peak ca. 1970 CE just before the increasing trend of planktonic diatoms, suggesting a lake/catchment disturbance likely related to the ski resorts constructions.

### The uniqueness of the great acceleration

Both L_flux_ and TOC_flux_ indicate unique patterns in the six lakes since 1850 CE and particularly during the last decades, suggesting changes in the depositional regimes. Despite the numerous CPs identified by CumSum methodology, most of them occurred in the mid to late twentieth century and at the end of the LIA (ca 1850 CE). Other CPs are associated with dam construction—MA and UR, 1920–1940—, a LIA phase in MA (the only lake with a direct connection with a small ice accumulation) and documented medieval deforestation in some low altitude lakes (SI, AC, UR). As we discussed before, prior to the twentieth century, periods of higher sediment input in the high-altitude Pyrenean lakes would be related to wetter and/or higher seasonality climate phases during the LIA and increased human pressure (twelfth to thirteenth centuries and late 1800s–early 1900s) (Figs. 3 and 4).

GAM analyses show the most important change in isotope signatures in the twentieth century in most lakes (CR, MA, SI, UR), except in AC that occurred at the end of the LIA and MA with no model adjustment. CumSum showed CPs in 1975 CE in CR, SI and SA and a little bit later (2000 CE) in MA. MA and AC have CPs at the end (1880) and the mid nineteenth century, respectively. GAM analyses showed an earlier CP for AC and CumSum for SI and CR (1452, 1782 CE) coinciding with CPs identified with other proxies. The analyses for the C/N record show more CPs prior to the twentieth century than for the other time series, but still most occurred in the mid to late twentieth century (CR, UR and AC) and in the late nineteenth century (SA and SI). Some of the variability in CPs could be attributed to the age uncertainty associated with the Bayesian age models. Biological evidence suggests that the increase in organic carbon accumulation during the last decades has been driven by increased productivity (Fig. S.7.A–C).

During the last century, a common feature of the lake records is the rapid increase in L_flux_, and both techniques identified CPs. This supports an intensification of surface processes (erosion, run-off) in the watersheds. Similar trends have been described in other settings with reduced recent human disturbance in the watersheds (Great Plains in the US; New Zealand), and they have been ascribed to higher dust deposition^54,55^. Dust could be a contributing factor in Pyrenean lakes as Saharan dust fluxes in the southern Pyrenees are the highest in the Iberian Peninsula^56^. In the case of the Pyrenean mountains, the documented trends of overall decrease in the duration of the snowpack, earlier snowmelt and the increasing frequency of winter rain events^45^ could be potentially responsible for increasing erodibility and runoff in alpine basins, conducive to higher sediment delivery to the lakes.

The TOC fluxes during the last decades are the largest in the records (Figs. 2 and 6) and both techniques identified most CPs during the last 200 years (Figs. 3 and 4). GAM analyses for lakes located at lower altitudes show main CPs during the twentieth century (SI, ca. 1950, UR, early 1900; SA 1950; AC ca 1910–20). In CR the main CP occurred earlier, and MA data could not be adjusted to a GAM model. The CumSum analysis also found earlier CPs (800–1200 and 1700–1800 CE in SI; 800–1100 and 1500–1800 in MA, 1700–1800 in SA). Some of them correlate with warmer phases during the Medieval Climate Anomaly and others during periods of higher soil watershed disturbance during medieval and modern times.

The individual uncertain age PCA (PC_AU_) for the four variables used in this study (δ^13^C_OM_, C/N, L_flux_ and TOC_flux_) show similar patterns and the % of variance explained by each PC1 was between 54 and 80% (Figure S.11). The structure of these PC1 versus age plots was similar to the general PC1_AUorg_ y PC1_AUlit_ and confirmed that the main changes occurred during the mid nineteenth century and mid twentieth century. For organic accumulation (PC1 TOC_flux_ and PC1 δ^13^C_OM_) the periods with the largest deviations occurred during the early and mid to late twentieth century and for sediment delivery (PC1 C/N and PC1 L_flux_) during the mid nineteenth century (end of LIA) and late twentieth century, although variability was larger.

The similarities in sites located at higher (CR) and lower (AC) altitude and with different bedrock geology (carbonate *vs* siliceous) supports global processes in lake ecology as main factors explaining TOC_flux_ and shifts in diatom assemblages during the GA (Figs. S.2A–C and S.7.A–C). A similar recent rapid increase on the planktonic component in another Pyrenean lake (Redon) has been correlated with higher air temperatures at the end of summer and more stable stratification^5^. Similar increases in planktonic diatoms have been related to climate warming^57^, and increasing nutrient availability^4,5,58^. Studies in other lakes in the central Pyrenees showed an increase in atmospheric P and N deposition during the last decades^59^. Higher atmospheric CO_2_, temperatures, nitrogen and phosphorus deposition all increase primary productivity^4,7^. Both, longer and more stable summer stratification and the observed increase in lithogenic mobilization from lake catchment would change lake nutrients availability for phytoplankton growth^23^. The decrease in C/D ratio (Chrysophyte/Diatoms ratio) and the increase in BioSi observed in lake CR reinforce the idea of an increase in the lake trophic state. Other factors, as faster C burial favoring C preservation due to higher Lflux and incomplete organic matter decomposition near the sediment–water interface, may have amplified the effects of increased bioproductivity and resulted in overestimation of recent TOC_flux_,

Hence, higher L_flux,_ TOC_flux,_ and planktonic diatoms in high altitude lakes identify the GA as a unique period with higher bioproductivity and sediment delivery caused by the synergistic effects of increasing temperatures, higher erodibility, and nutrient availability.

## Conclusions

High altitude Pyrenean lakes show the greatest changes in sediment delivery and organic carbon accumulation since 1850 and 1950 CE over the last 1200 years.

Prior to the twentieth century, higher lithogenic fluxes occurred during periods of increased human pressure (early medieval times, late nineteenth century) and wetter phases of the LIA. Lakes at lower altitudes (AC, SA, SI) show higher TOC_flux_, lower δ^13^C_OM_ and C/N during medieval times, after 1850 CE and an accelerating trend since 1950s, all indicative of increase in carbon burial and bioproductivity. Main periods of change in higher altitude lakes occurred at the same periods, but with variable trends and smaller ranges. The δ^13^C_OM_ trends in alkaline lakes suggest additional sources of Dissolved Inorganic Carbon during the Great Acceleration from the watershed carbonate formations.

Increased sediment fluxes to the lakes during the last decades may be related to changes in seasonality, leading to longer ice-free periods and higher erodibility of rainfall versus snow precipitation. Increased organic accumulation is driven by higher algal productivity, primarily controlled by higher temperatures and longer ice-free and growing season, although higher atmospheric input of nutrients could also be a significant factor. The exponential increase in lithogenic and organic carbon fluxes in all lakes since the mid twentieth century is a unique feature in the last 1200 years.

## Methods

### Monitoring and field work

Lakes were surveyed for bathymetric maps, sediment traps were deployed and limnological parameters (temperature, pH, conductivity, oxygen content) were measured in mid Summer and early Autumn. Short sediment cores were retrieved with a gravity UWITEC corer, split, imaged and logged with a Geotek Multi-Sensor Core Logger (MSCL). Watersheds were sampled to characterize vegetation, soils and sediments (C/N and isotope composition) and the drainage network, geology, geomorphology and soils were mapped from available data and field surveys.

### Sedimentology

The definition of sedimentary facies has followed four main criteria^60^: (1) grain size; clays: 0.01–2.0 µm, silts: 2.0–63.0 µm and sands: > 63.0 µm^61^. (2) Main components as of organic matter, silicates and carbonates calculated from the elemental data of organic and inorganic carbon^62^: % Carbonates = (100.09 × TIC)/12.01; % Organic matter = % TOC × 1724 and % silicates = 100 − % Carbonates − % MO. (3) Sedimentological features such as color, lamination (massive, banded, laminated or finely laminated), and grain size. (4) Geochemical composition, based on XRF scanner and ICP.

TOC fluxes were calculated as:

$${\text{TOC}}_{{{\text{flux}}}} = {\text{ density }} \times {\text{ Sedimentation rate}} \times \, \% {\text{TOC}}.$$

Lithogenic Flux were calculated as:

$${\text{L}}_{{{\text{flux}}}} = {\text{ density }} \times {\text{ SR }} \times \, \left( {{1}00 \, - \, \% {\text{TOC }} - \% {\text{TIC}}} \right). \, \left[ {\text{SR was obtained from the age models}} \right].$$

### Geochemistry

Total carbon (TC), total inorganic carbon (TIC) were analyzed at 1 cm resolution in the sediment cores and in watershed and sediment trap samples at the IPE-CSIC (Zaragoza, Spain) using a LECO SC144 DR analyser. For TIC analysis, organic matter was previously removed at 460 °C in a muffle furnace for 300 min. The Total Organic Carbon (TOC) was calculated as the difference between TC and TIC.

The δ^13^C_OM_ in bulk organic matter were analyzed with a FlashEA 1112 (ThermoFinnigan) coupled with an interface ConfloII (ThermoFinnigan) to a mass spectrometer of isotopic ratios Deltaplus (ThermoFinnigan) at the University of A Coruña, Spain.

Quantitative chemical analyses were performed in the composite sequences at a lower resolution (every 1 cm, 150 mg weight sample) with an ICP-OES 720-ES (Varian) at the Experimental Station El Zaidin—CSIC (Granada, Spain). Elements measured by ICP-OES were Al, As, Ca, Cd, Co, Cr, Cu, Fe, K, Li, Mg, Mn, Mo, Na, Ni, P, Pb, S, Se, Si, Sr, Ti, V, Zn. Samples were digested with HCl and HNO_3_ (1:1:3 de H_2_O:HCl:HNO_3_) in a UltraWAVE (Milestone) at 220 °C during 15 min.

Samples for grain size analyses were heated 24 h at 80 °C in 3% hydrogen peroxide to eliminate organic matter and were measured with a MasterSizer 2000 at the IPE-CSIC laboratories. Smear slides and thin sections were used to define sedimentary facies using an optical microscope.

Biogenic silica (BioSi) was measured every 4 cm using the wet-alkaline leaching technique^63^ after carbonates and organic matter were removed by HCl 1 M and peroxide. Then, BioSi was leached with Na_2_CO_3_ 2 M and solution separated from the remaining sediment by centrifugation and bicarbonate was neutralized with HCl. BioSi, as dissolved silicate, was measured by the molybdate blue colorimetric method using an AutoAnalyser Technicon II^64^ at the IIM-CSIC.

Photosynthetic pigments in bulk sediment were analyzed at the University of Valencia, Spain, with three successive pigment extractions measured with a spectrophotometer Beckman DU 640; the chlorophyll and the pheophytin in the sediment were calculated according the equations described in^65^.

Sediment samples for diatom analysis were processed using hydrogen peroxide (33% H_2_O_2_) and HCl (2 ml 1 M) and mounted in Naphrax on a microscope slide following the method described in^66^. A minimum of 300 diatoms valves were identified per slide, and simultaneously we counted chrysophyte cysts to calculate C/D ratio (Chrysophyte/Diatoms ratio) using a Zeiss Axio Imager A1 microscope (Carl Zeiss Inc., Germany) equipped with a 100× objective (Zeiss Plan-Apo 1.4 numeric aperture) and differential interference contrast optics at 1000 magnification. Diatoms concentration in sediment samples was estimated by adding a known number of microspheres^66^. Diatom identifications were based mainly on^67^ and taxonomic references therein, with the currently accepted diatom names following the Algabase database (https://www.algaebase.org/).

### Age models

Age models were obtained combining ^210^Pb-^137^Cs techniques performed at St. Croix Watershed Research Station (Minnesota, USA) using gamma ray spectrometry and AMS ^14^C dating (Table S.3). We used Bayesian statistics to simulate the accumulation rate of the different lakes with the Bacon v2.5.8 Bayesian age-depth model^32^ implemented in the geoChronR package v1.1.6^33^.

### Statistical analysis

We calculated a “Compositional PCA'' (PC_comp_) including compositional (TOC, TIC), geochemical (elements measured in ICP) and grain-size data (% sand) (Fig. S.2.A–C) and used the first PC1_comp_ (explaining 53% of the variance) as an indicator of the lithogenic composition of the sediments. We used the geoChronR package v1.1.6^33^ to implement two “age-uncertain PCA”^41^ that were applied to two groups of variables: (i) TOC_flux_, C/N and δ^13^C_OM_ and (ii) L_flux_ and PCA_comp_. GeoChronR uses a binning procedure to achieve this across multiple ensembles^33^ and a probabilistic PCA (PPCA) approach using the pcaMethods package^68^. For the “diatom PCA”, we used a Principal Components Analysis (PCA) based on Hellinger transformation^69^ of diatoms counts (only diatoms with relative abundance higher than 1% and with at least two occurrences across the entire record) to extract the main components of variability in CR and AC. The PCA was performed using the R package Vegan (2.5-7)^70^.

A cumulative sum (CumSum) of deviations of the mean^40^ was applied to the time series of δ^13^C_OM_, C/N, L_Flux_ and TOC_flux_. A CumSum is the sequential sum of the successive elements of a variable after removing from each temporal observation the mean of the time series and plotting the cumulative sum of residuals. A positive slope of the CumSum indicates an above-average tendency in the data and a negative slope represents a below-average tendency, meanwhile changes in the slope of the cumulative sum curve from positive to negative or vice versa indicate phases for which the mean of the data is higher or lower than the mean of the original time series^40^. Not all the identified Change Points (CPs) (Fig. 4) have similar weight, and to evaluate their significance we have taken into consideration the change in slope and sign as well as the number of elements close to the CPs, as this technique works better for time series with more than 50 elements and ours contain between 38 and 83 points. The CumSum was programmed in R and the source code is available upon request.

We applied the change point (CP) method proposed by^38^ based on the use of GAMs (Generalized Additive Models)^39^ (Fig. 3) This method fits a nonlinear trend to the time series and then estimates the first derivative of the function that represents this trend. It also estimates a confidence interval (95%) around the fitted function that is used to determine statistically significant temporal changes, i.e. periods of significant change are identified as those time points where the simultaneous (lower and upper) confidence interval on the first derivative does not include zero. These analyses were performed using the R packages “mgcv”^71^ and “gratia”^72^ following the supplementary material provided in^38^.

## Supplementary Information

Supplementary Information.

## Data Availability

The datasets used or analyzed during the current study are available URL: 10.5281/zenodo.7953552.
