# Supplementary material for: Unprecedented recent regional increase in organic carbon and lithogenic fluxes in high altitude Pyrenean lakes
Source: Sci Rep. 2023 May 26;13:8586. doi: 10.1038/s41598-023-35233-1 (PMC10220012; doi:10.1038/s41598-023-35233-1)
Supplement: Supplementary file 1 — Supplementary Information. [file 41598_2023_35233_MOESM1_ESM.pdf]

Unprecedented recent regional increase in Organic Carbon and Lithogenic fluxes in high altitude Pyrenean lakes

Data Availability

The datasets generated during and/or analysed during the current study are available in the ZENODO repository [https://doi.org/10.5281/zenodo.7953552].

Supplementary Material

Watershed Geology and Topography

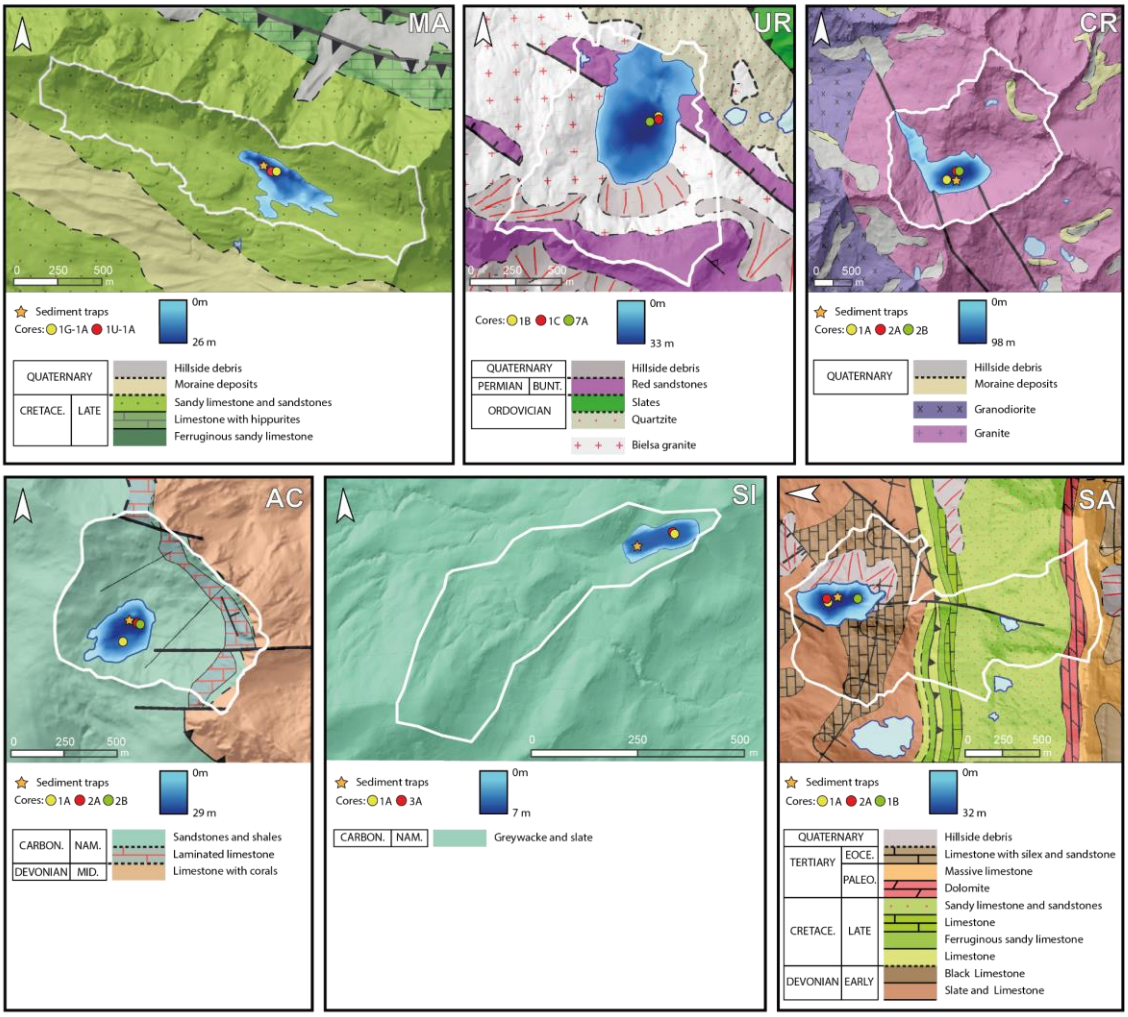

Fig. S.1. Watershed geology and topography and lake bathymetry, location of the cores and sediment traps. (Figure made with Adobe Illustrator CS6: https://www.adobe.com/es/products/illustrator.html)

Limnological features of the lakes

Lake	Latitude	Longitude	Lake area (Km^2)	Catchment (Km^2)	Lake perimeter (Km)	Altitude (masl)	Max. Depth (m)	Lake volume (m^3)	Chlorophyll (µg/l) sup.	Trophic character	Conductivity (µS/cm)		Alkalinity (ppm)	
											sup.	max. deep	sup.	max. deep
Marboré (MA)	42,69546	0,04009	0,088	0,957	1,9486	2600	26	1379134	0,1436	Ultra-oligotrophic	69	77	58,6	57,5
Acherito (AC)	42,8797	-0,70705	0,058	0,569	1,0662	1877	29	583689	4,35	Mesotrophic	110	119	81,6	82,8
Sabocos (SA)	42,6926	-0,25741	0,096	2,317	1,364	1900	32	1183798	0,0818	Ultra-oligotrophic	169	209	117,4	141,6
Urdiceto (UR)	42,66524	0,2803	0,296	1,051	2,3276	2364	33	3557078	0,6118	Ultra-oligotrophic	35	35	34,6	36,5
Cregüeña (CR)	42,63766	0,62288	0,445	3,403	4,384	2633	98	20392626	0,315	Ultra-oligotrophic	14	14	15,9	13,9
Sierra (SI)	42,74221	-0,41502	0,013	1,66	0,46	2022	7	27081	2,71	Mesotrophic	18	19	22,3	23,2

Table S.1. Lake basin and watersheds main features and limnological characteristics of the lakes.

Sedimentology, sedimentary facies and sediment composition

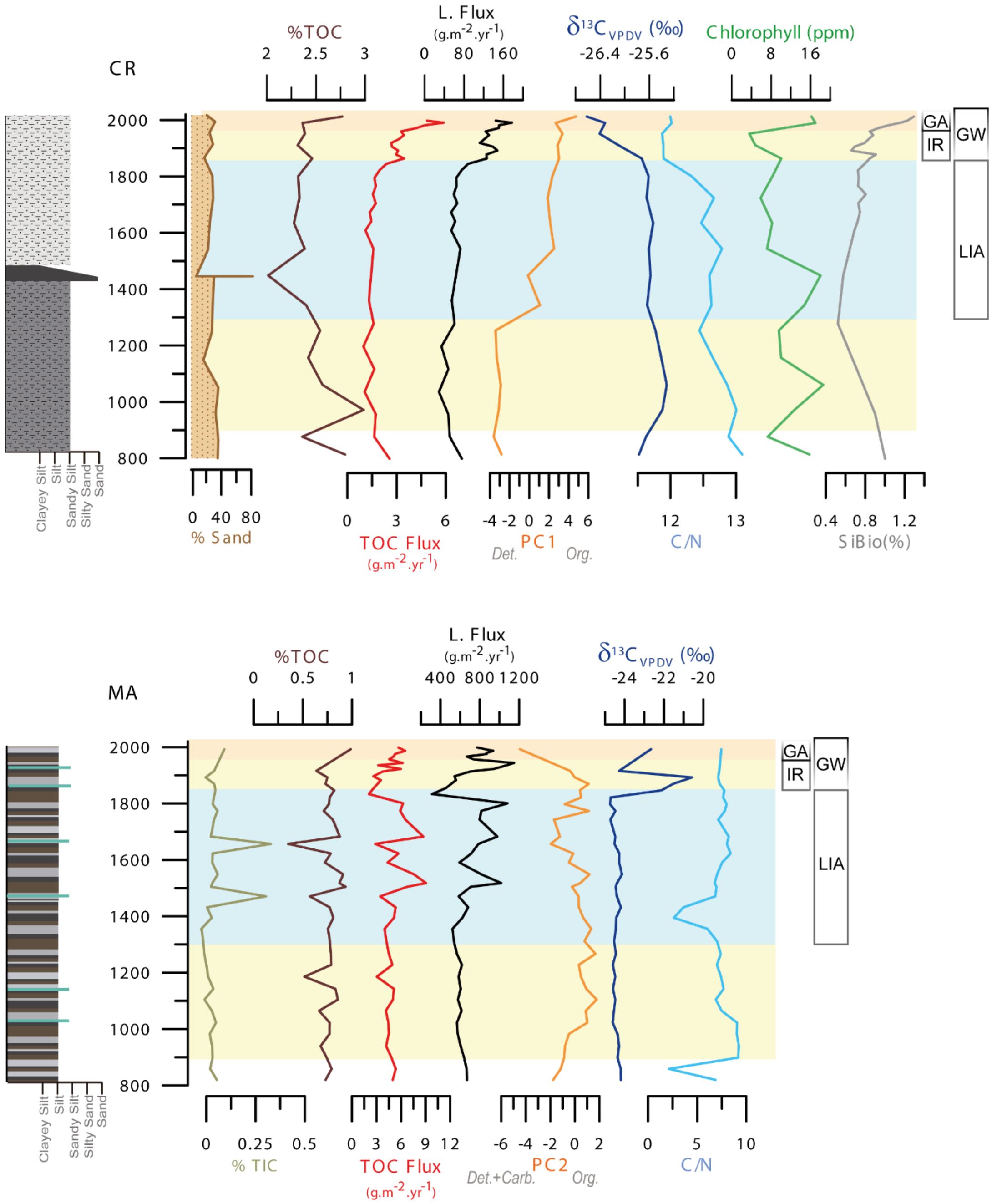

Fig. S.2.A Sedimentological facies (see description in table S.2), % sand (light brown), %TOC (dark brown), %TIC (green), TOC flux (red), Lithogenic flux (black), $PC1_{comp}$ (orange), $\delta^{13}C_{OM}$ (dark blue), C/N (light blue), chlorophyll (green) and biogenic silica (gray) from Cregueña and Marboré. Climate phases (Little Ice Age, LIA and Recent Global Warming, GW) and global change phases (Industrial Revolution, IR and Great Acceleration, GA) are also indicated.

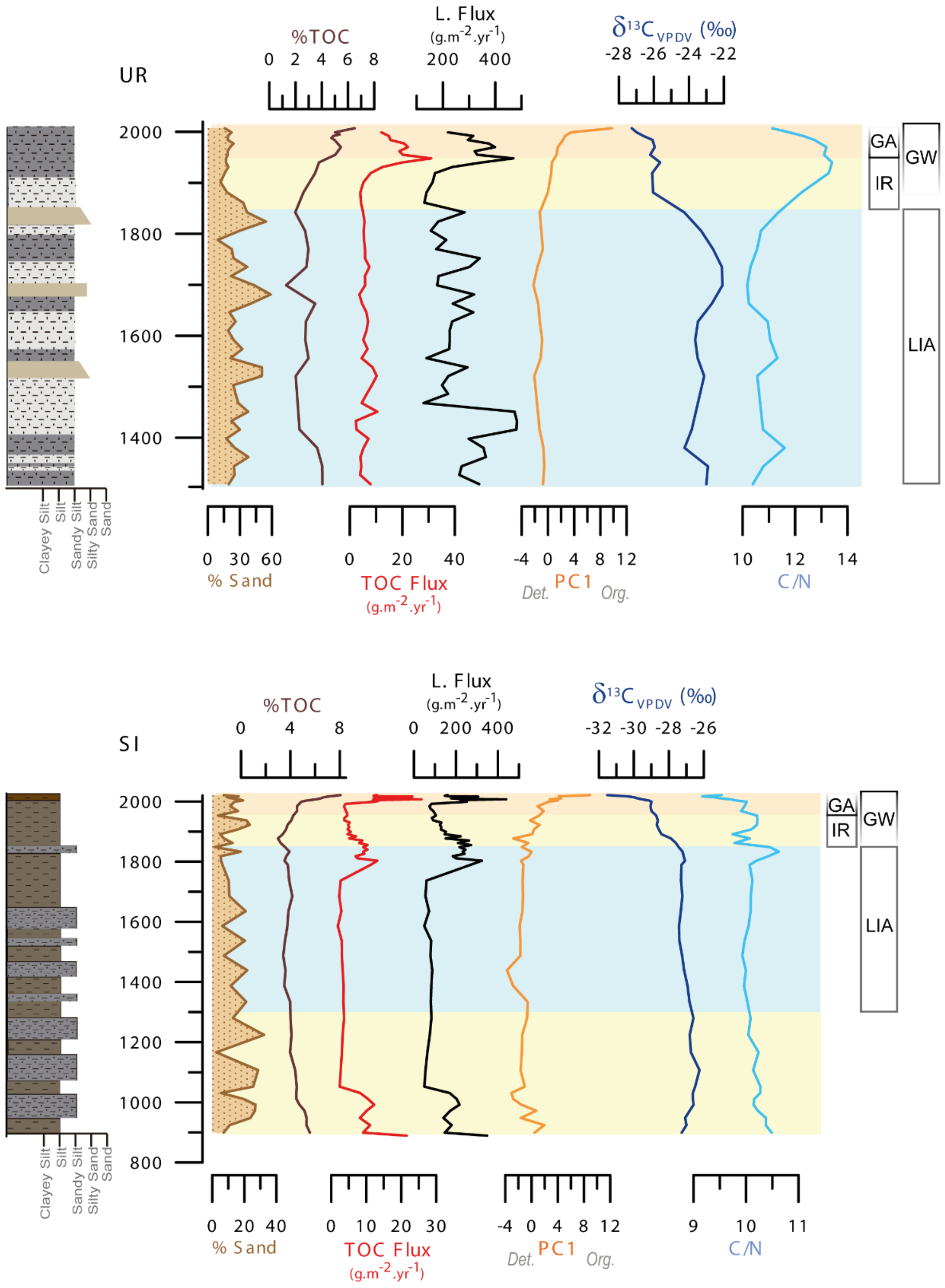

Fig. S.2.B Sedimentological facies (see description in table S.2), % sand (light brown), %TOC (dark brown), TOC flux (red), Lithogenic flux (black), $PC1_{comp}$ (orange), $\delta^{13}C_{OM}$ (dark blue) and C/N (light blue) from Urdiceto and La Sierra. Climate phases (Little Ice Age, LIA and Recent Global Warming, GW) and global change phases (Industrial Revolution, IR and Great Acceleration, GA) are also indicated.

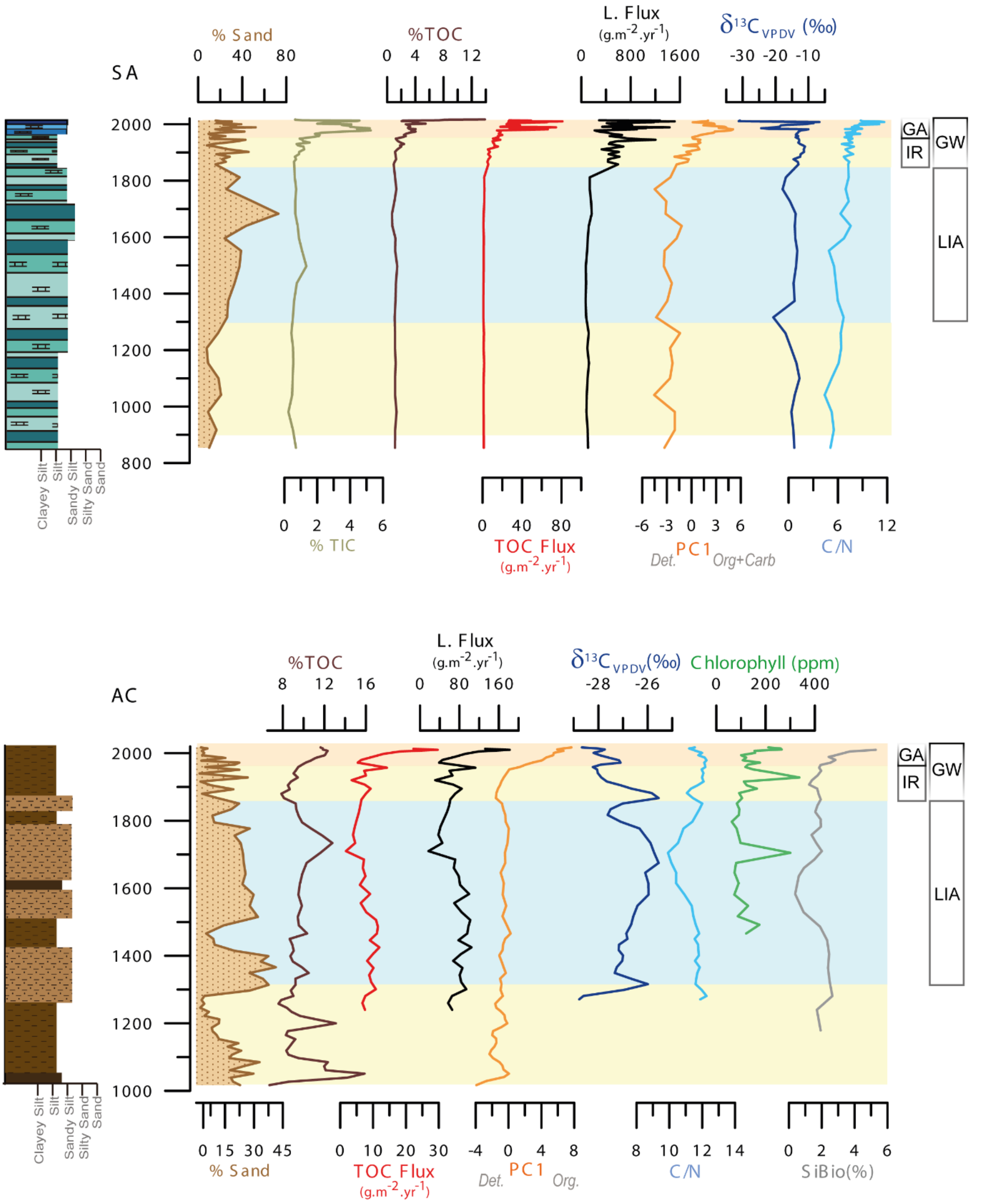

Fig. S.2.C Sedimentological facies (see description in table S.2), % sand (light brown), %TOC (dark brown), %TIC (dull green), TOC flux (red), Lithogenic flux (black), $PC1_{comp}$ (orange), $\delta^{13}C_{OM}$ (dark blue), C/N (light blue), chlorophyll (green) and biogenic silica (gray) from Sabocos and Acherito. Climate phases (Little Ice Age, LIA and Recent Global Warming, GW) and global change phases (Industrial Revolution, IR and Great Acceleration, GA) are also indicated.

	Facies	Legend	Composition	Granulometry	Description	Depositional Interpretation	Lakes
Siliciclastic	1		≥95% silicates	Sandy silt	Brown to dark brown sandy silt, slightly banded (1-3 cm)	Clastic deposition in distal areas of the lake during periods of relatively higher (sandy facies) and lower (silty facies) energy, likely related to variable runoff and water and sediment delivery from the watershed	CR, UR
	2		90 to 95% silicates	Sandy silt	Greyish brown to dark browns sandy silt		CR, SI, UR
	3		91 to 95% silicates	Silt	Banded greyish brown silt		SI
	4		> 90 % silicates	Silty sand	Massive light brown silty sand		UR
	5		> 90 % silicates	Clayey silt	Pink clay silt		UR
	6		≥95% silicates	Sands to clayey silt	Fining upward layer (6 cm) from dark brown sand at the base to brown clay silt at the top	Turbidite deposited after the Ribagorza earthquake (1373 CE).	CR
	7		≥95% silicates	Silts and silty clay	Silt and silty clay, laminated to banded (1 mm to 1,5cm), with alternating brown, white and gray layers	Rhythmite: Clastic deposition controlled by seasonal to pluri-annual changes in run-off and sediment delivery from the watershed	MAR
Organics	8		10 to 20% OM	Sandy silt	Massive light brown sandy silt with abundant OM	Deposition in distal areas of the lakes with higher organic productivity	AC, UR
	9		10 to 25% OM	Silt	Massive grayish brown silt with abundant OM		AC, SI
	10		10 to 30% OM	Sandy silt to silt	Dark grayish brown sandy silt, banded and with abundant OM		AC
Carbonataded	11		5 to 10% carbonate<br>10 to 25% OM	Sandy silt	Finely laminated brown sandy silt with presence of carbonates and abundant OM	Deposition in distal areas of the lake including sediment from the watersehd, carbonates and organics from the littoral areas (charophyte meadows)	SA
	12		15 to 45% carbonate<br>4 to 9% OM	Sandy silt	Finely laminated brown sandy silt with abundant carbonates		SA
	13		25 to 45% carbonate<br>4 to 9% OM	Sandy silt	Dark brown sandy silt, laminated and rich in carbonates		SA
	14		2 to 25% carbonate<br>≤ 5% OM	Sandy silt	Sandy brown sils, laminated to banded, with the presence of carbonates.		SA

Table S.2. Facies Description. Sedimentary facies were identified following the classification of lacustrine sediments proposed by TMI tools (https://tmi.laccore.umn.edu/). We have defined three major facies categories: 1) Siliciclastic, the most common, with more than 90% silicate components, 2) Organic, with more than 10% OM (between 10 and 30%), 3) Carbonate-bearing (> 2% carbonate).

Age models

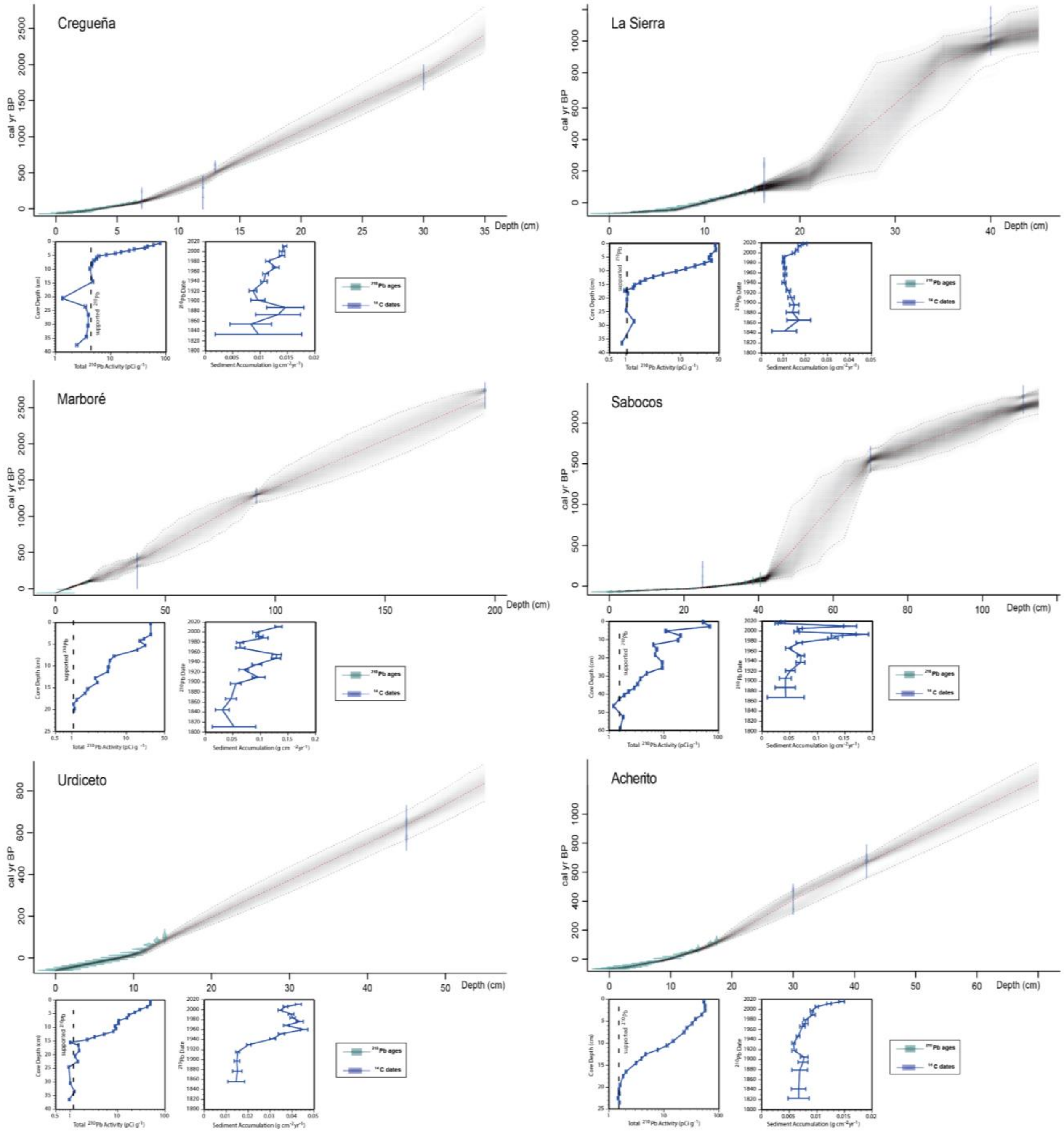

Fig. S.3. Depth-Age Models of the studied lakes, Total ^{210}Pb activity (pCi g^{-1}) and Sediment Accumulation (g cm^{-2} yr^{-1}) are shown in the insets

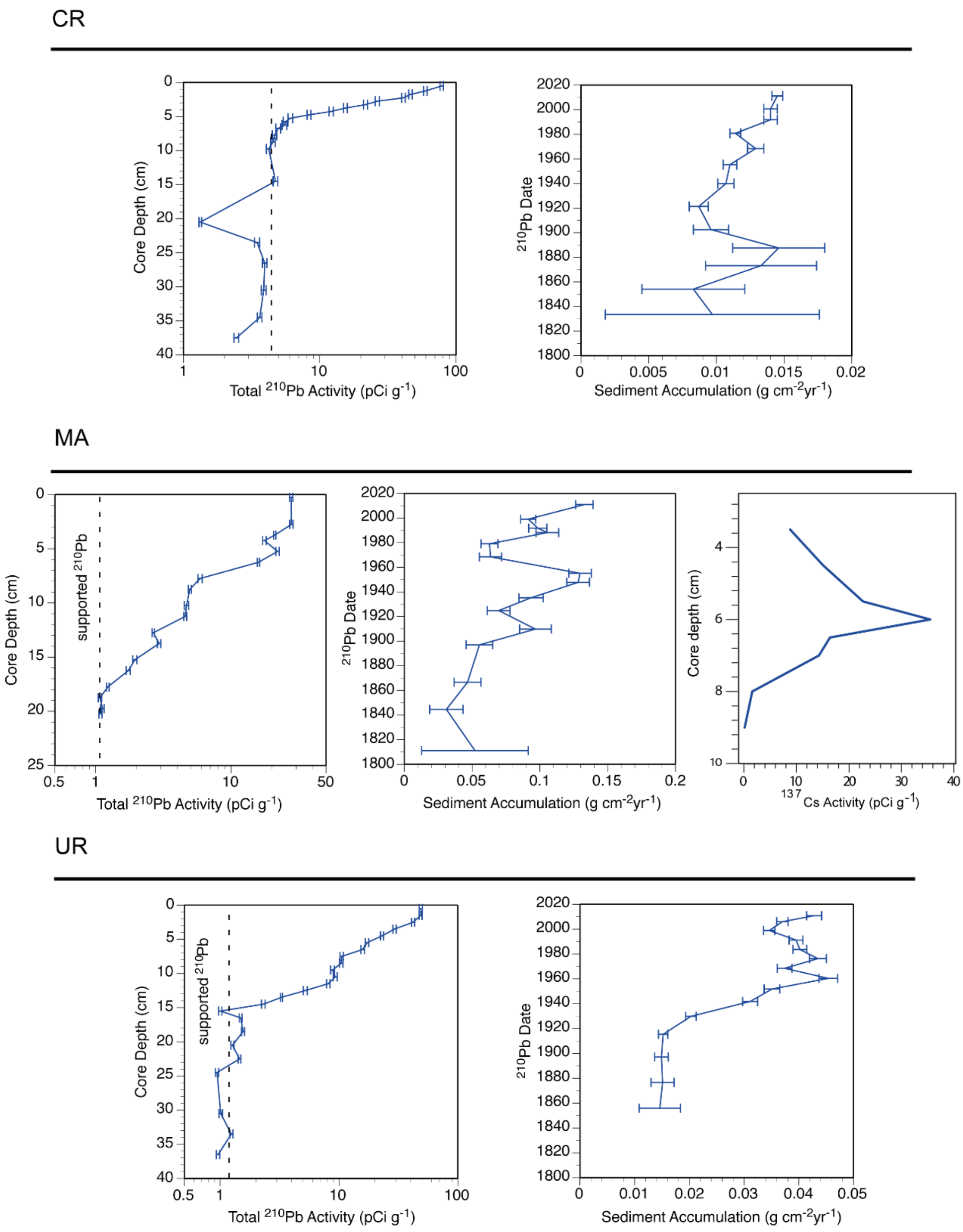

Fig. S.4.A Total ^{210}Pb activity (pCi g^{-1}) and sediment accumulation (g cm^{-2} yr^{-1}) of CR, MA and UR lakes. In the case of Marboré, the results of the total ^{137}Cs activity are also included.

SI

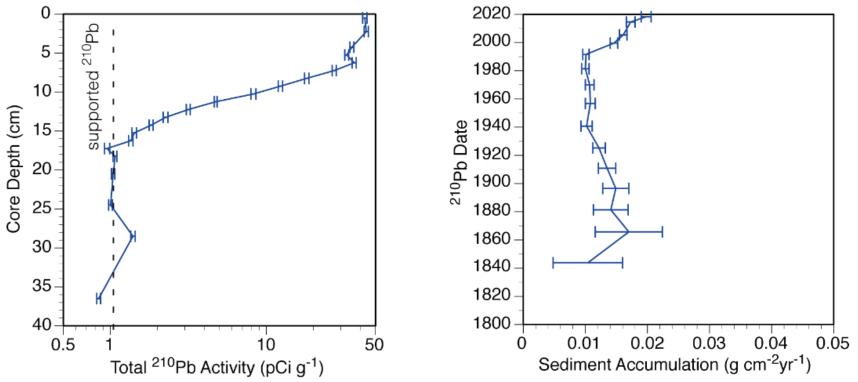

SA

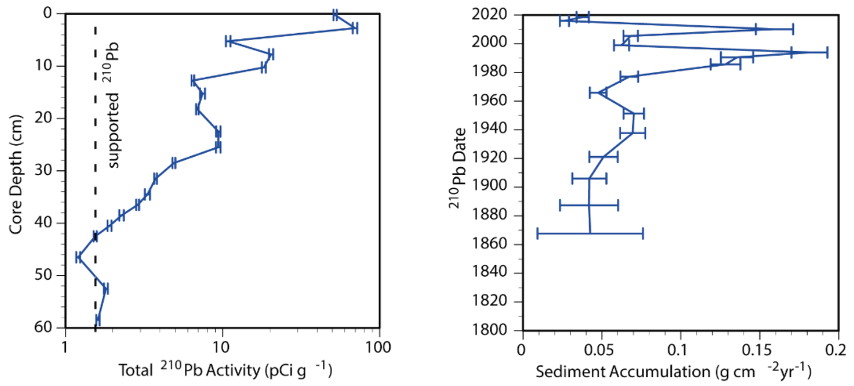

AC

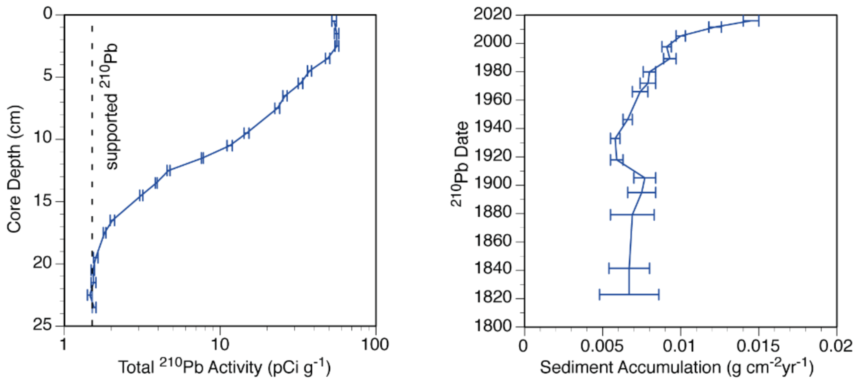

Fig. S.4.B Total ^{210}Pb activity (pCi g^{-1}) and sediment accumulation (g cm^{-2} yr^{-1}) of SI, SA and AC lakes.

Lake	DirectAMS code	Submitter ID	Sample type	Fraction of modern		Radiocarbon age	
				pMC	1 σ error	BP	1 σ error
AC	D-AMS 028339	REP-ACH17-2A-1G 49-50 cm	plant material	95.36	0.23	382	19
	D-AMS 028340	REP-ACH17-2A-1G 61-62 cm	plant material	90.97	0.33	760	29
SI	D-AMS 028338	REP-SIR17-3A-1G 45-46 cm	sediment (bulk)	86.59	0.23	1157	21
CR	D-AMS 028010	CRE17-2A-1G 9-10 cm	sediment (bulk)	85.04	0.27	1302	26
	D-AMS 028011	CRE17-2A-1G 14-15 cm	sediment (bulk)	83.74	0.27	1425	26
	D-AMS 028009	CRE17-1A-1G 22-23 cm	sediment (bulk)	78.17	0.25	1978	26
	D-AMS 028012	CRE17-2A-1G 40-41 cm	sediment (bulk)	66.14	0.22	3321	27
SA	D-AMS 037572	SAB13-1B-1G-1 (A), 2-3 cm	sediment (bulk)	85.01	0.41	1305	39
	D-AMS 037573	SAB13-1B-1G-1 (A), 47-48 cm	sediment (bulk)	68.86	0.24	2997	28
	D-AMS 037574	SAB13-1B-1G-1 (A), 88-89 cm	sediment (bulk)	63.87	0.22	3601	28
UR	D-AMS 024321	BIE-URD12-1B-1G	plant material	92.11	0.44	660	38
MA	D-AMS 001189	MAR11-1A-1U-1 cm 39-41	sediment (bulk)	73.13	0.23	2514	25
	D-AMS 1217-204	MAR11-1A-1U-2 cm 53-56	sediment (bulk)	63.79	0.22	3611	28

Table S.3. AMS ^{14}C dates included in the age models of the lake sequences

PCA Analyses

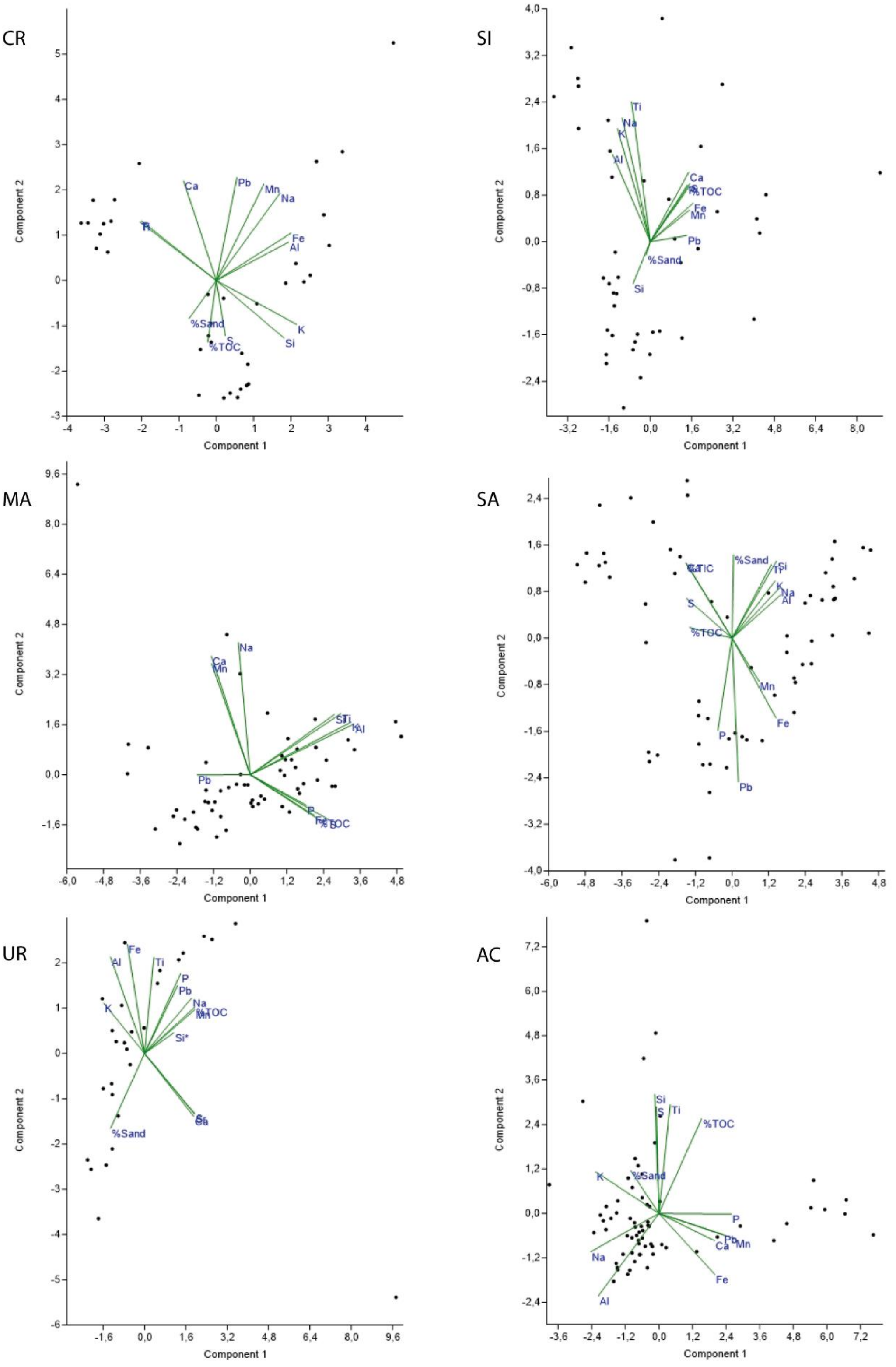

Fig. S.5. PC1 and PC2 components for the Compositional PCA (PC_{comp}) obtained from geochemical data (ICP), %TOC, %Sand and %TIC (in MA and SA). The PC1 of each lake explains between 53.5% and 37.5% of variance (SA 53.5%, SI 48.4%, AC 43.1%, CR 39.9%, MA 37.5%).

C/N and $\delta^{13}C_{OM}$

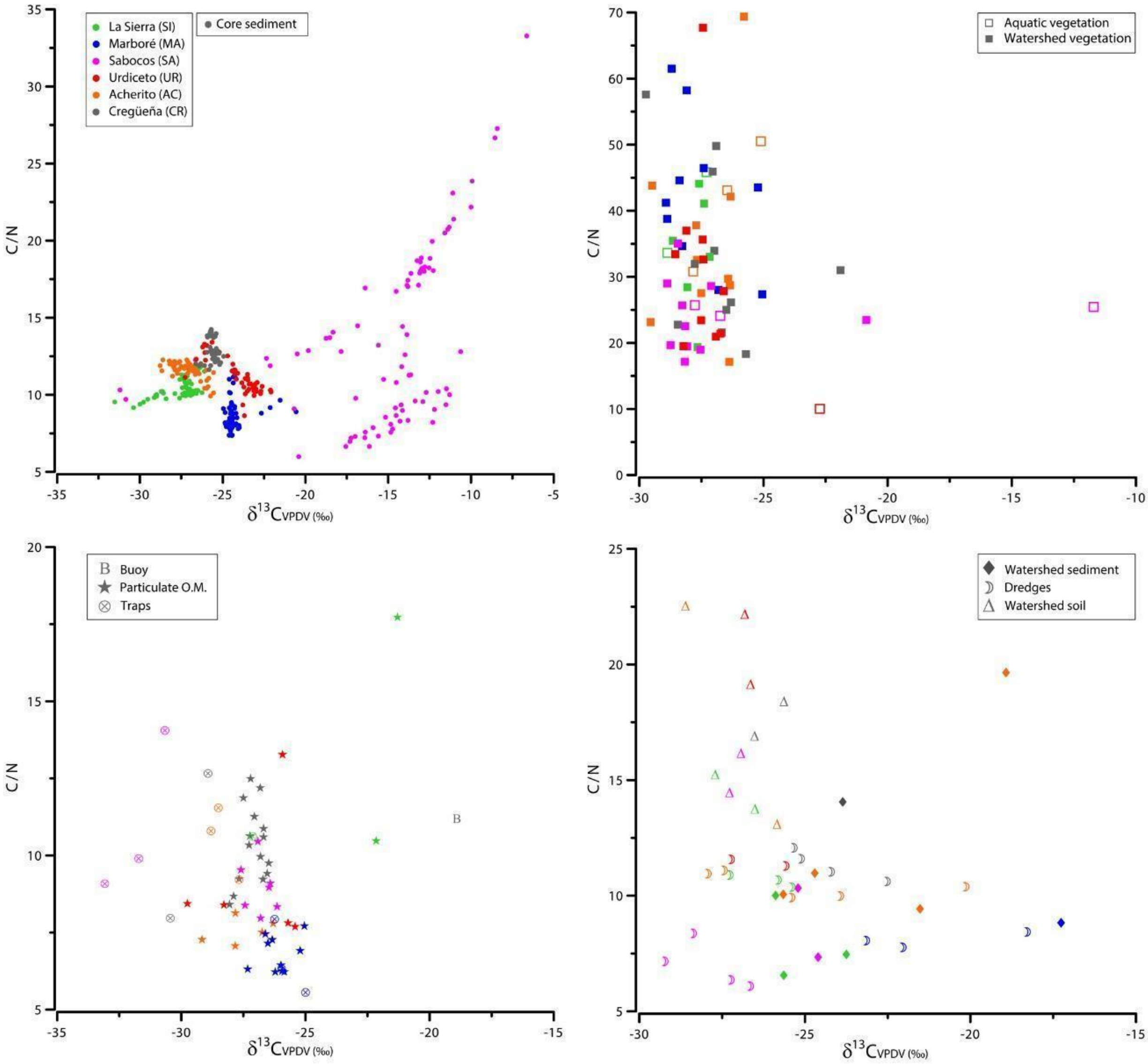

Fig. S.6. C/N vs $\delta^{13}C_{OM}$ for each lake considering sample type for each lake (core sediment, buoy, POM, sediment traps, dredges, aquatic vegetation) and for each watershed (vegetation, sediment and soil).

Diatom stratigraphy

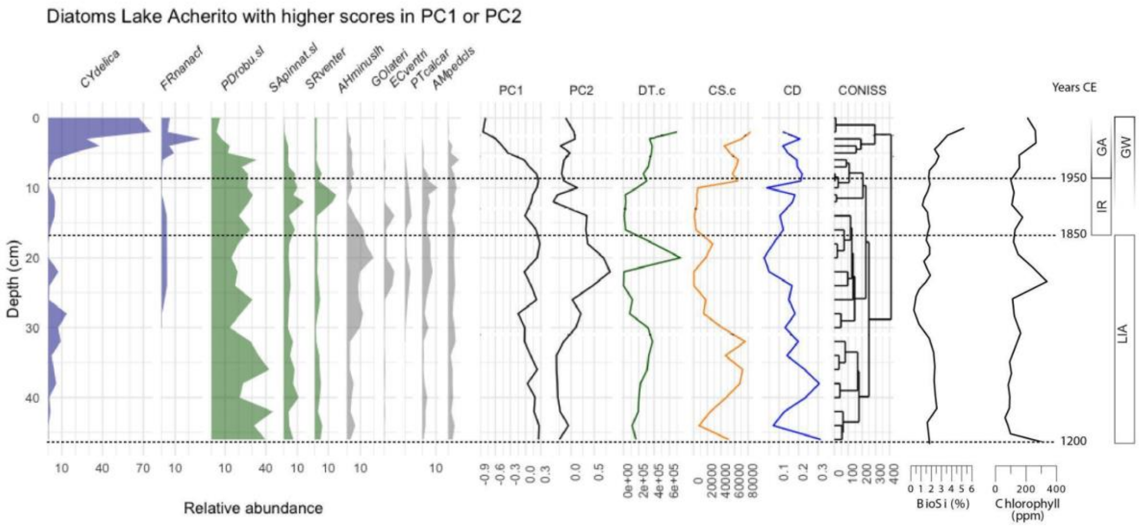

Fig. S.7A. Diatom stratigraphy, biogenic silica (%), and chlorophyll of the Acherito (AC) sequence. The climate phases (Little Ice Age, LIA and Recent Global Warming, GW) and the global change phases (Industrial Revolution, IR and Great Acceleration, GA) are also indicated.

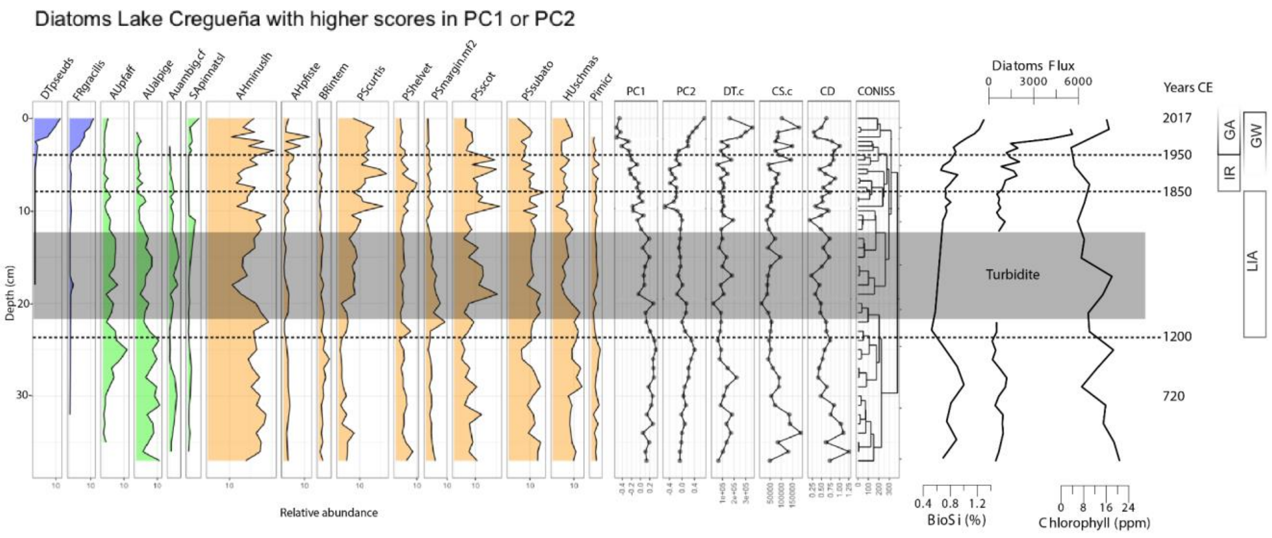

Fig. S.7.B. Diatom stratigraphy, diatom flux, biogenic silica (%), and chlorophyll (ppm) of the Cregueña (CR) sequence. The climate phases (Little Ice Age, LIA and Recent

Global Warming, GW) and the global change phases (Industrial Revolution, IR and Great Acceleration, GA) are also indicated

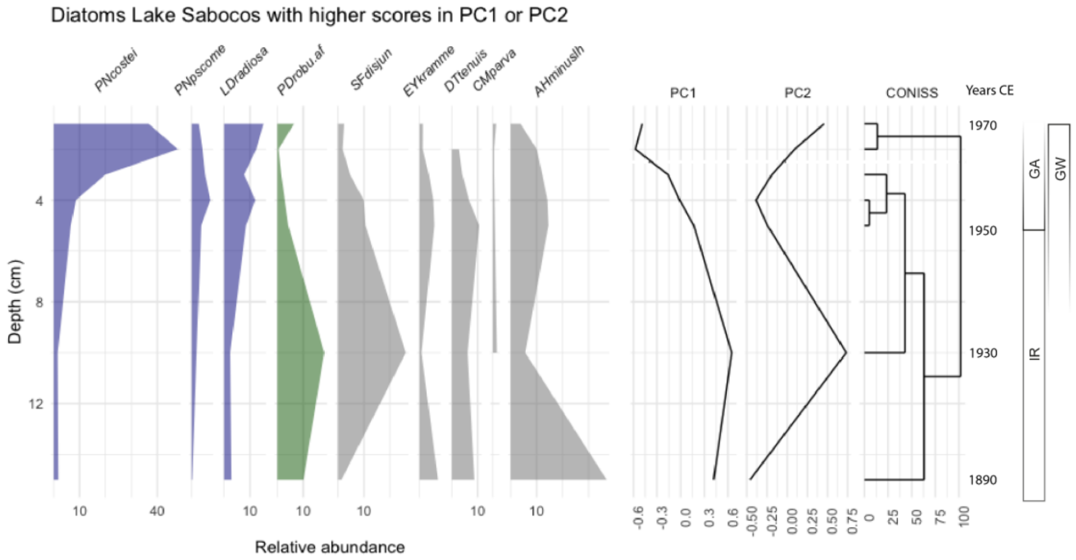

Fig. S.7C. Diatom stratigraphy of the Sabocos (SA) sequence. The climate phases (Recent Global Warming, GW) and the global change phases (Industrial Revolution, IR and Great Acceleration, GA) are also indicated.

Lithogenic and TOC fluxes, C/N and $\delta^{13}C_{OM}$

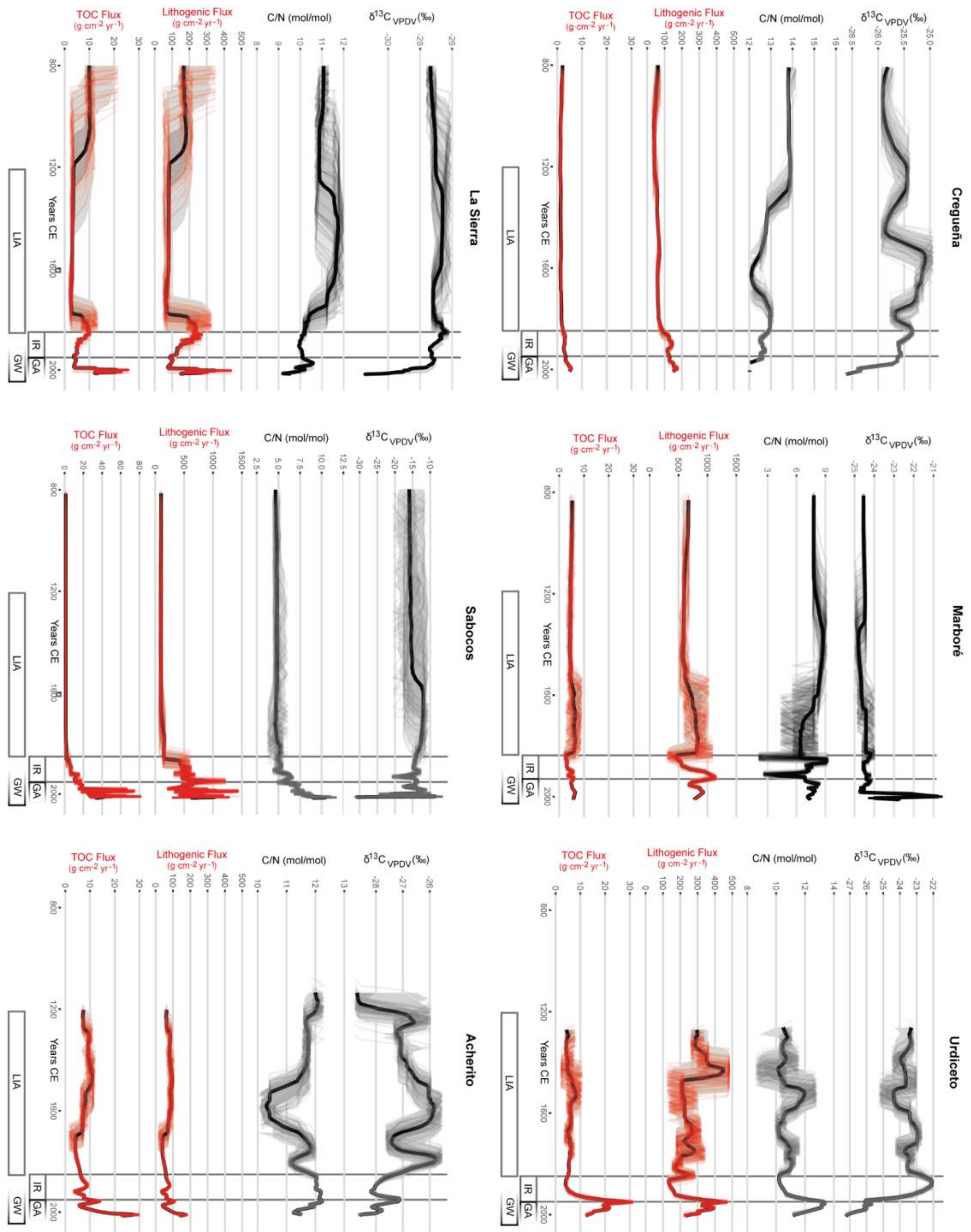

Fig S.8. Lithogenic and TOC fluxes (red), C/N and $\delta^{13}C_{OM}$ (black) for the last 1200 years in the studied lake sequences (CR, MA, UR, SI). The climate phases (Little Ice Age, LIA and Recent Global Warming, GW) and the global change phases (Industrial Revolution, IR and Great Acceleration, GA) are also indicated. The median estimate is shown in black, and the 50 % and 95 % highest-probability density regions are shown in

dark and light gray. Random age-uncertain ensemble members are shown in red (Lithogenic and TOC fluxes) and gray (C/N and $\delta^{13}C_{OM}$).

Change Point Analysis

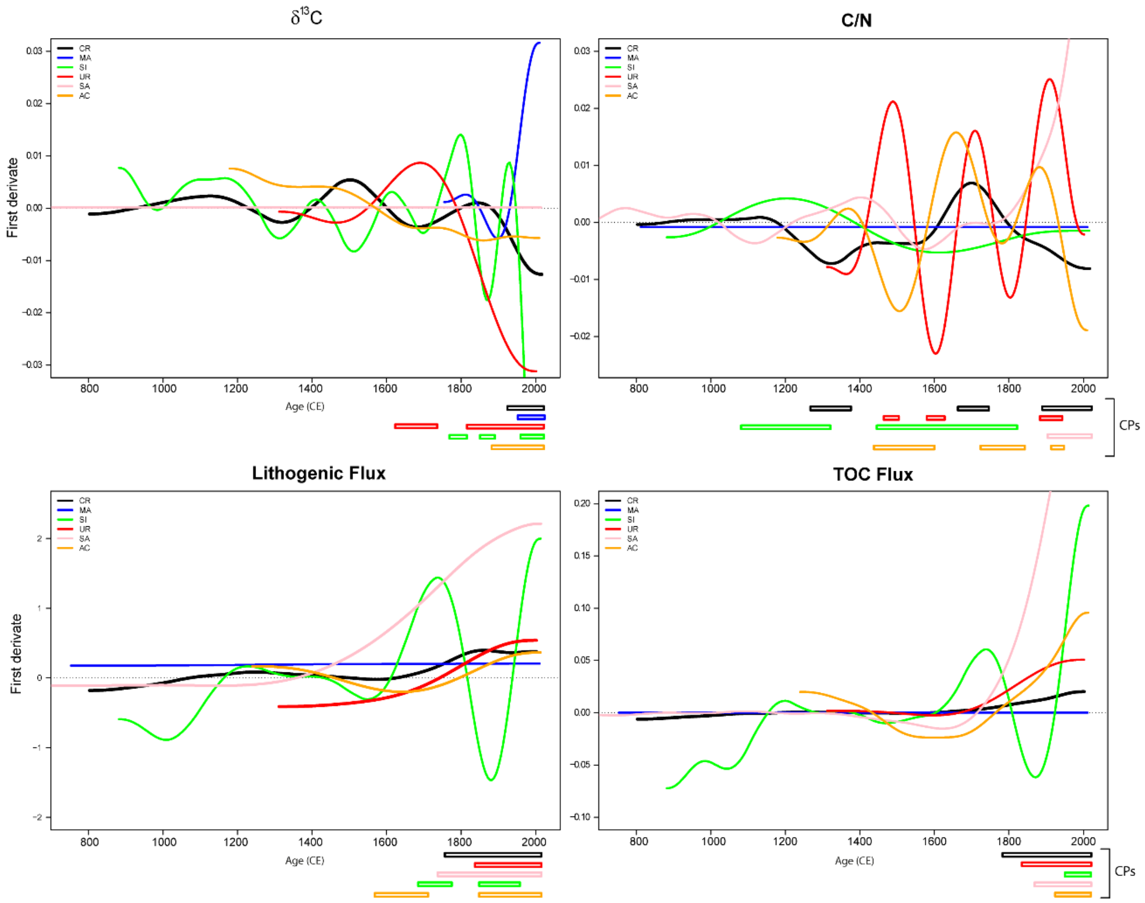

Fig S.9. Estimated first derivatives of the GAM trends fitted to L flux, TOC flux, C/N and $\delta^{13}C_{OM}$ time series and location of the significant temporal changes. The 95% simultaneous confidence intervals of the first derivatives are in Fig. S.10.A, B).

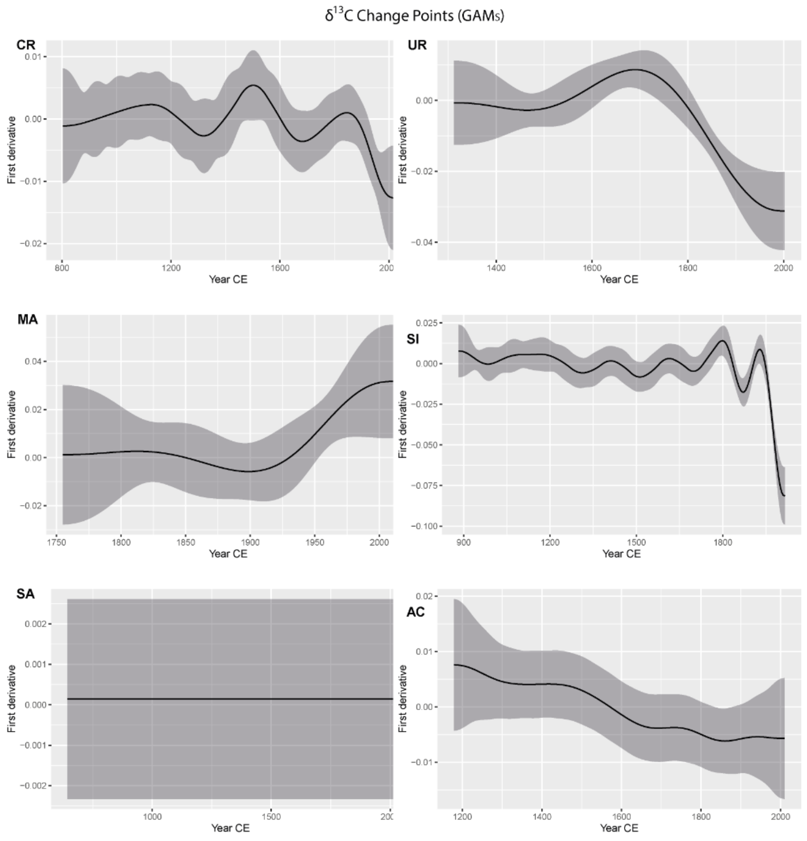
δ13C Change Points (GAMs)
CR
UR
MA
SI
SA
AC
First derivative
Year CE

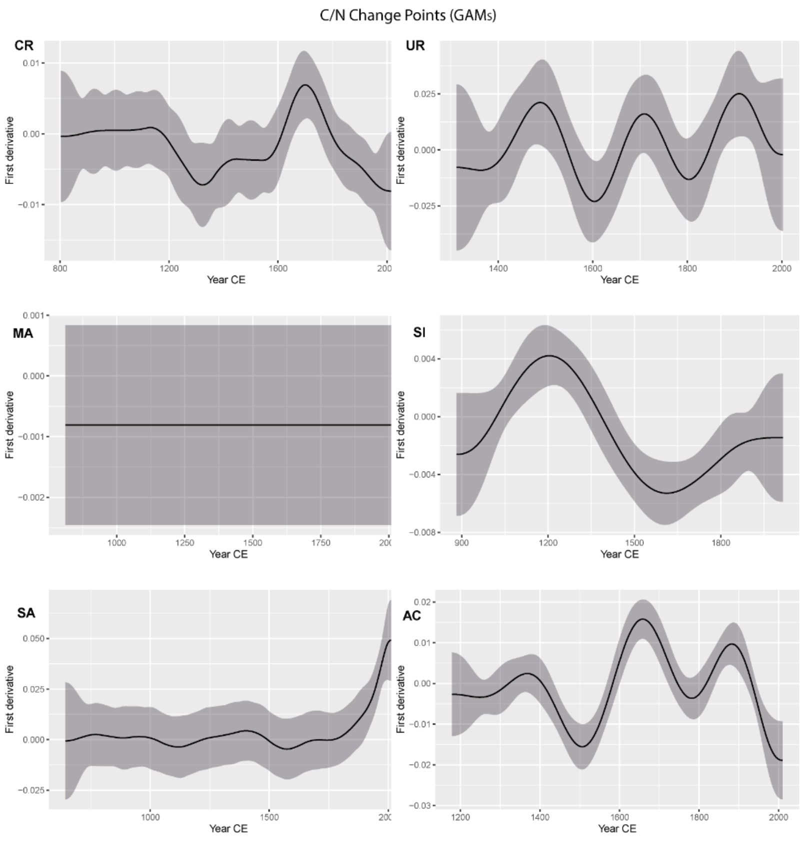
C/N Change Points (GAMs)
CR
UR
MA
SI
SA
AC
First derivative
Year CE

Fig. S.10A. Estimated first derivatives (black lines) and 95% simultaneous confidence intervals of the GAM trends fitted to $\delta^{13}C_{OM}$ and C/N time series. Where the simultaneous interval does not include 0, the models detect significant temporal change in the response.

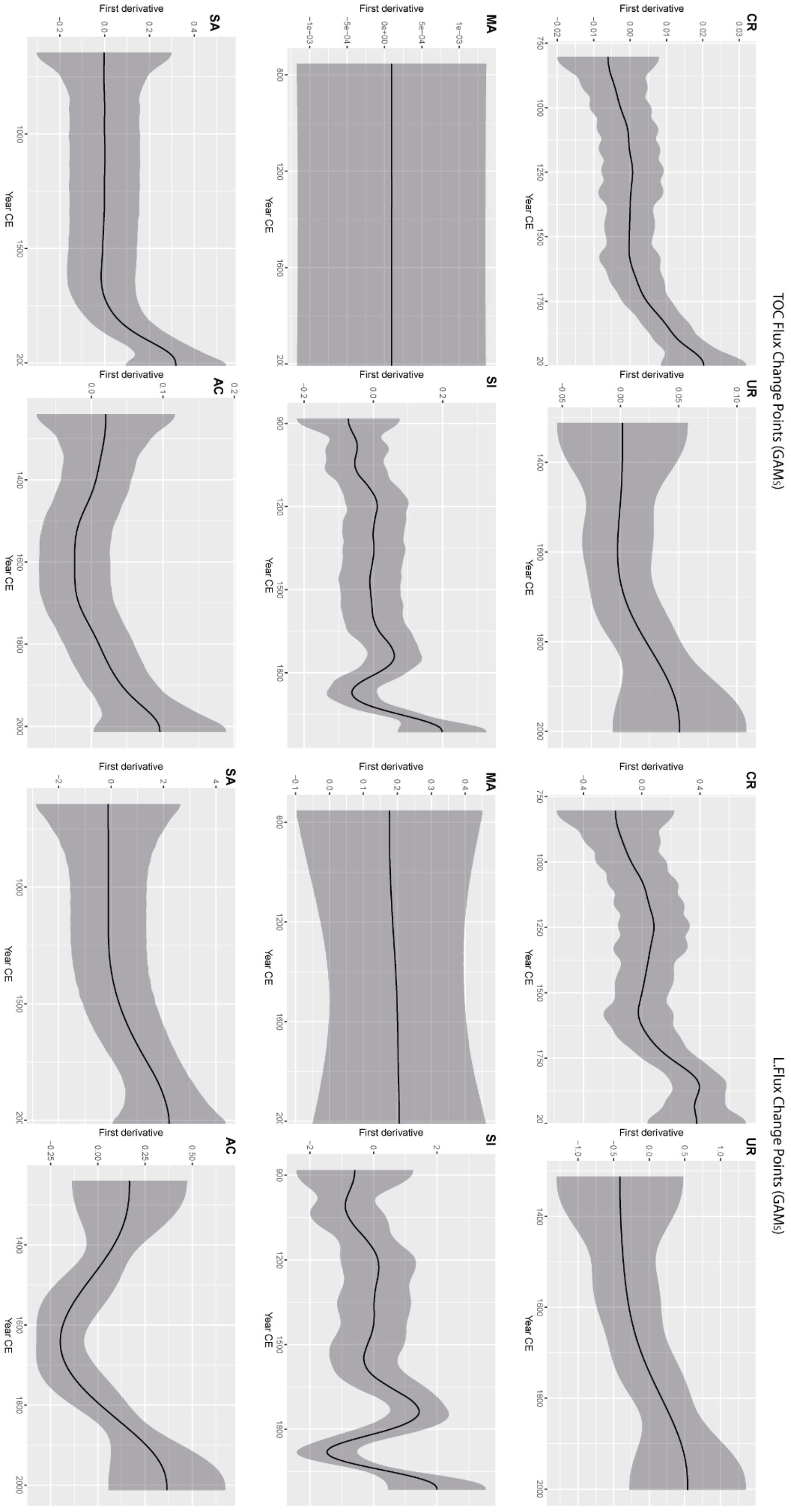

Fig. S.10B. Estimated first derivatives (black lines) and 95% simultaneous confidence intervals of the GAM trends fitted to LFlux and TOC flux time series. Where the simultaneous interval does not include 0, the models detect significant temporal change in the response.

Age- uncertainty PCA for individual variables

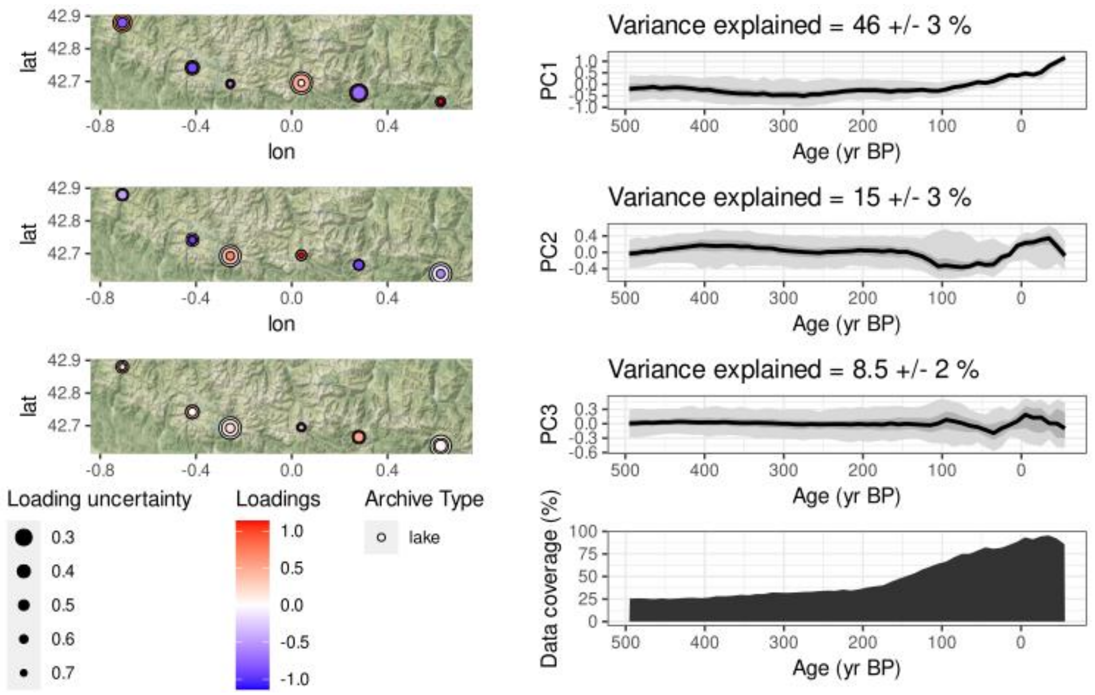

Figure S.11. Principal component analysis (PCA) for organic accumulation (all lakes).

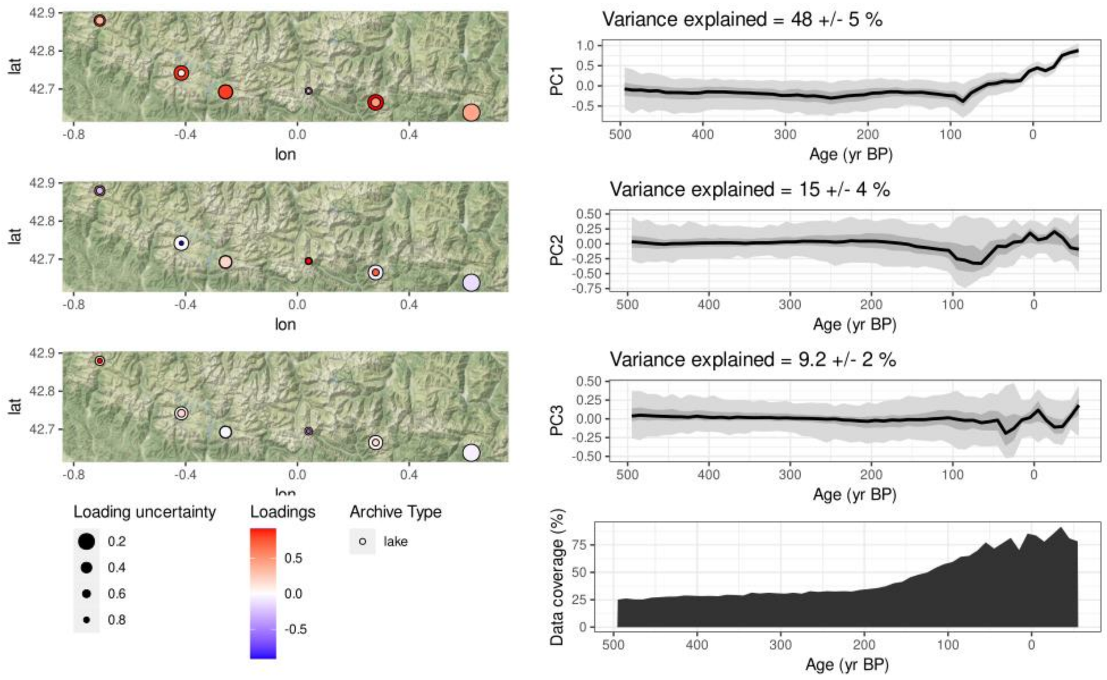

Figure S.12. Principal component analysis (PCA) for sediment delivery (all lakes).

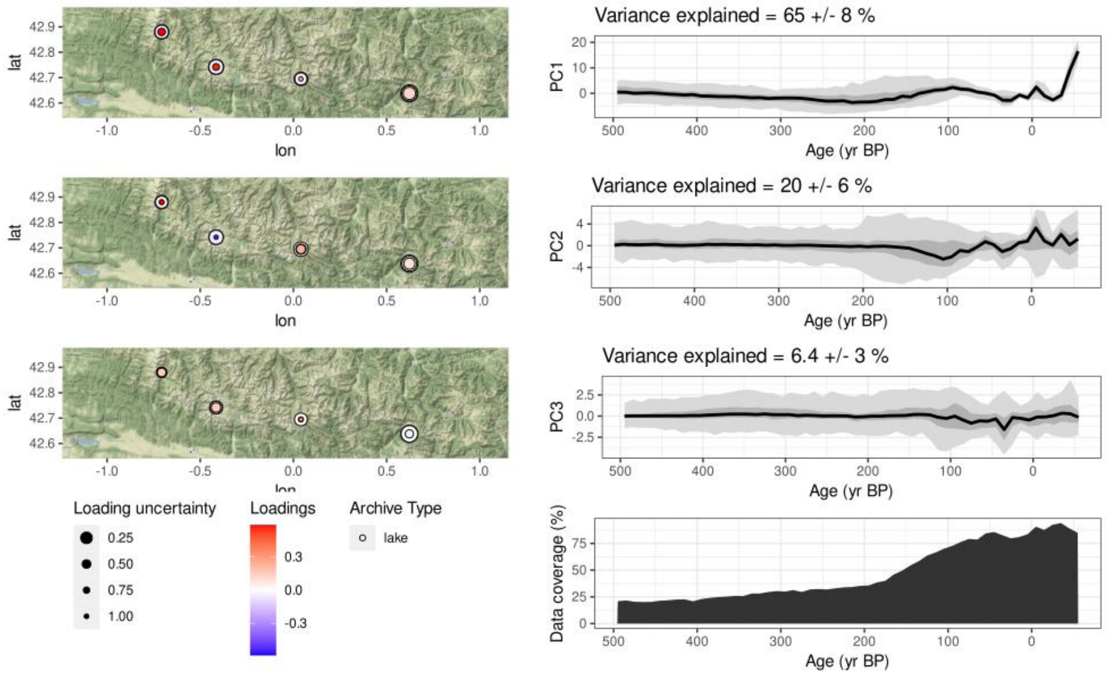

Figure S.13. Principal component analysis (PCA) for organic matter sources, excluding URD and SAB.

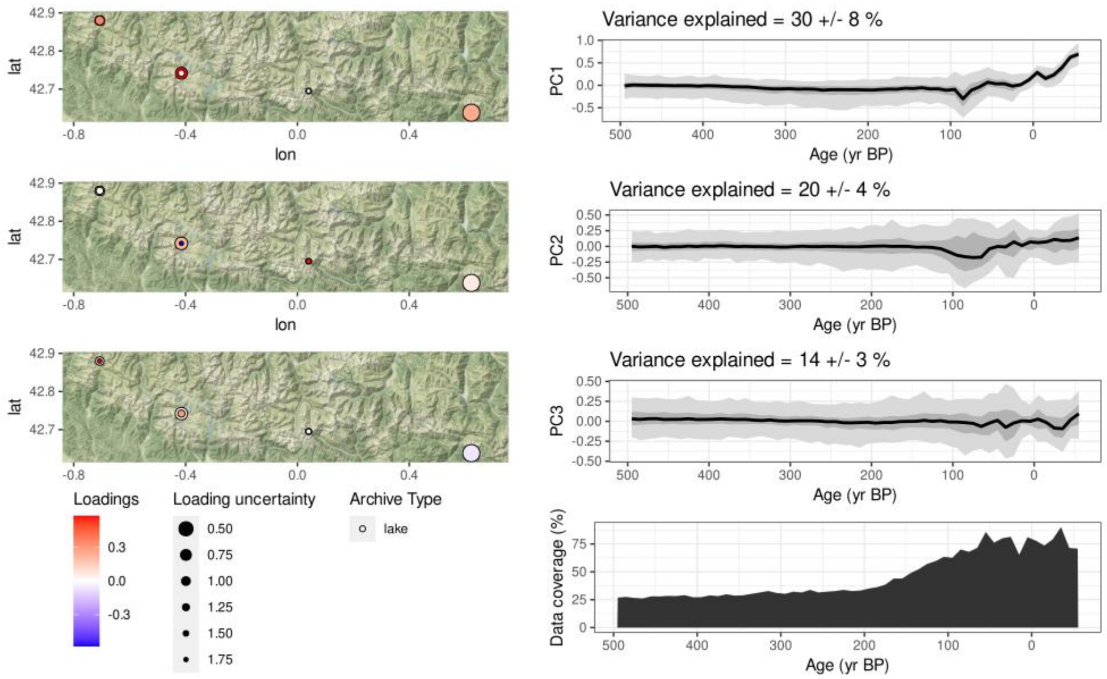

Figure S.14. Principal component analysis (PCA) for sediment delivery, excluding URD and SAB.

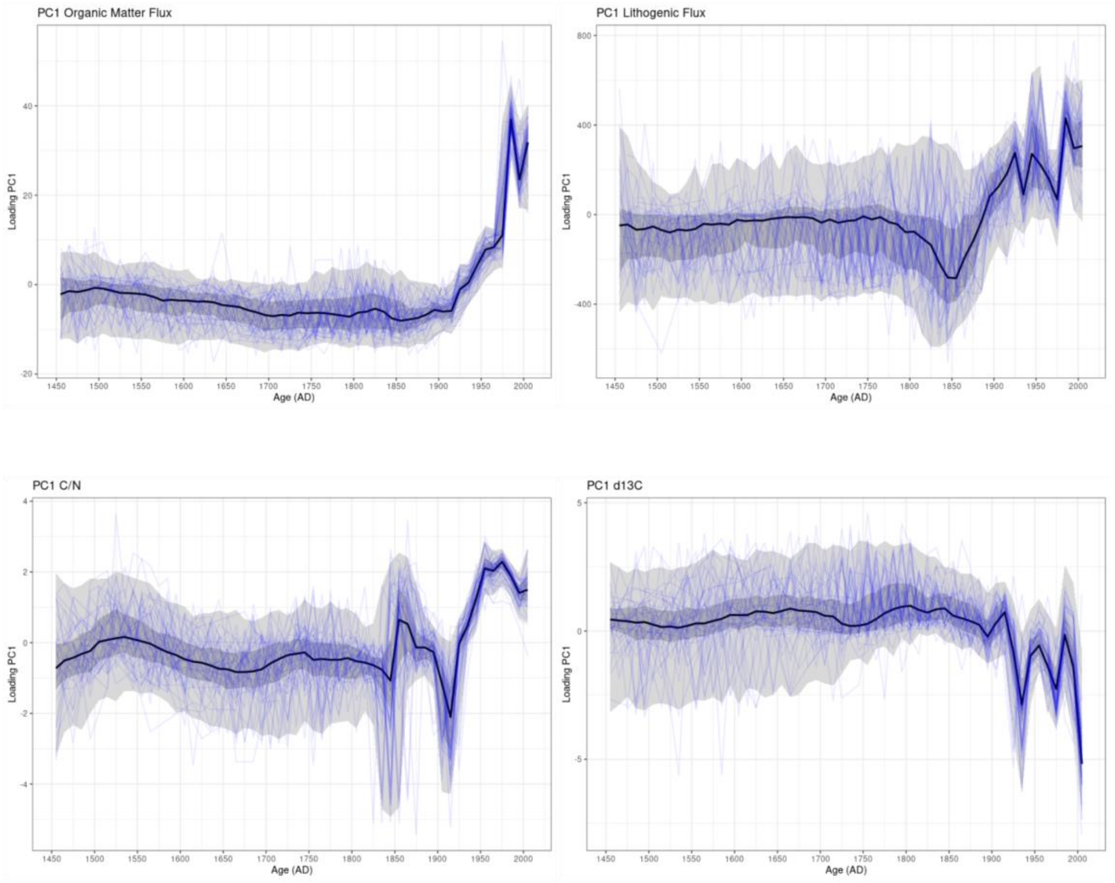

Figure S.15. First Principal Component (PC1) for PCA obtained for C/N, $\delta^{13}C_{OM}$, TOC fluxes and lithogenic fluxes, including all lakes. The median ensemble member is shown in black, with the 50 % and 95 % highest-density probability ranges shown in dark and light gray, respectively. The PC1 explained most of the variance for each variable: 80 % for TOC fluxes, 68 % for L flux, 58 % for $\delta^{13}C_{OM}$ and 54 % for C/N. Age-uncertain ensemble members are shown in blue.

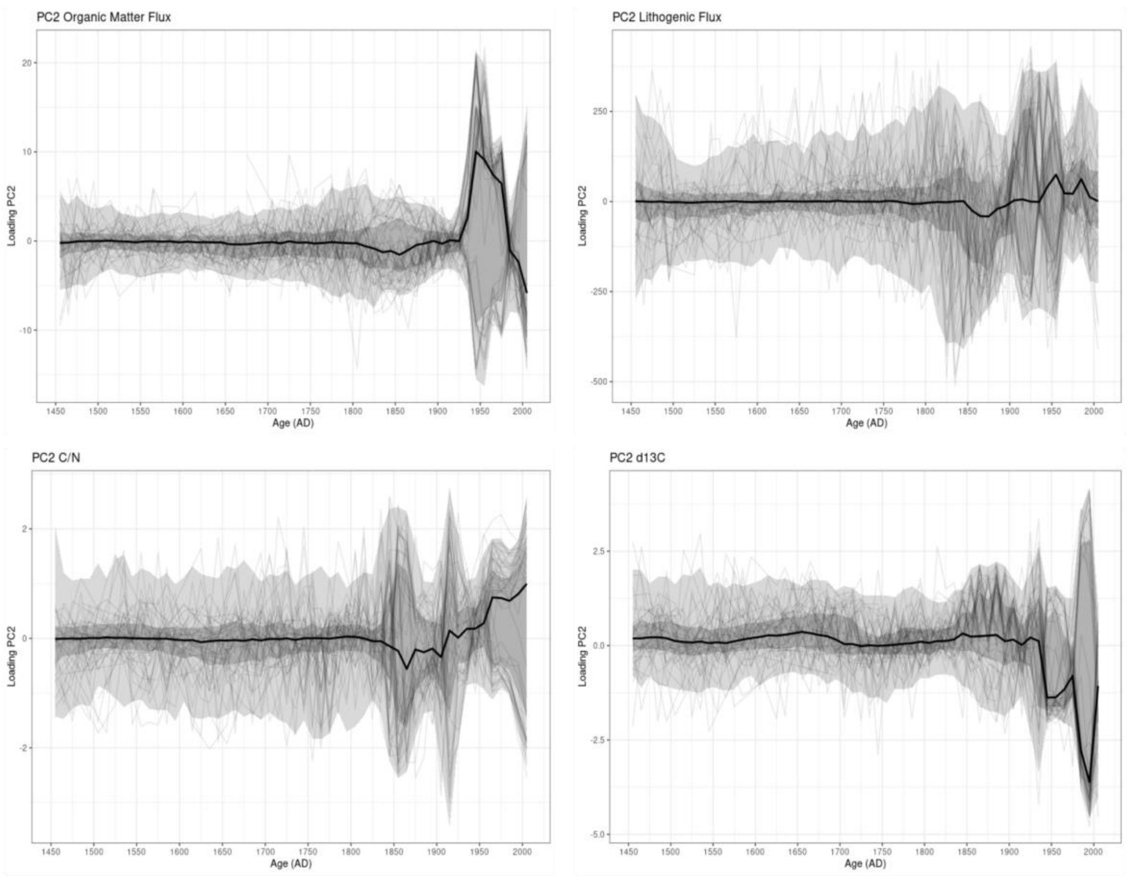

Figure S.16. Second Principal Component (PC2) for C/N, $\delta^{13}C_{OM}$, TOC fluxes and Lithogenic fluxes (all lakes). The median ensemble member is shown in black, with the 50 % and 95 % highest-density probability ranges shown in dark and light gray, respectively. Age-uncertain ensemble members are shown in gray.
